# Breast cancer cell-derived extracellular vesicles promote CD8^+^ T cell exhaustion via TGF-β type II receptor signaling

**DOI:** 10.1038/s41467-022-31250-2

**Published:** 2022-08-01

**Authors:** Feng Xie, Xiaoxue Zhou, Peng Su, Heyu Li, Yifei Tu, Jinjin Du, Chen Pan, Xiang Wei, Min Zheng, Ke Jin, Liyan Miao, Chao Wang, Xuli Meng, Hans van Dam, Peter ten Dijke, Long Zhang, Fangfang Zhou

**Affiliations:** 1 Institutes of Biology and Medical Science, Soochow University, Suzhou, China; 2 MOE Laboratory of Biosystems Homeostasis & Protection and Innovation Center for Cell Signaling Network, Life Sciences Institute, Zhejiang University, Hangzhou, China; 3 State Key Laboratory for Diagnostic and Treatment of Infectious Diseases, The First Affiliated Hospital, School of Medicine, Zhejiang University, Collaborative Innovation Center for Diagnosis and Treatment of Infectious Disease, Hangzhou, China; 4 Laboratory of Human Diseases and Immunotherapies, West China Hospital, Sichuan University, Chengdu, China; 5 The first affiliated hospital of soochow university, Suzhou, China; 6 Institute of Functional Nano & Soft Materials (FUNSOM), Jiangsu Key Laboratory for Carbon-based Functional Materials and Devices, Soochow University, Suzhou, China; 7 Department of Breast Surgery, Zhejiang Provincial People’s Hospital, Hangzhou, China; 8 Department of Cell and Chemical Biology, Oncode Institute, Leiden University Medical Center, Leiden, The Netherlands

**Keywords:** Metastasis, Cell signalling, Transforming growth factor beta, Immunosurveillance

## Abstract

Cancer immunotherapies have shown clinical success in various types of tumors but the patient response rate is low, particularly in breast cancer. Here we report that malignant breast cancer cells can transfer active TGF-β type II receptor (TβRII) via tumor-derived extracellular vesicles (TEV) and thereby stimulate TGF-β signaling in recipient cells. Up-take of extracellular vesicle-TβRII (EV-TβRII) in low-grade tumor cells initiates epithelial-to-mesenchymal transition (EMT), thus reinforcing cancer stemness and increasing metastasis in intracardial xenograft and orthotopic transplantation models. EV-TβRII delivered as cargo to CD8^+^ T cells induces the activation of SMAD3 which we demonstrated to associate and cooperate with TCF1 transcription factor to impose CD8^+^ T cell exhaustion, resulting in failure of immunotherapy. The levels of TβRII^+^ circulating extracellular vesicles (crEV) appears to correlate with tumor burden, metastasis and patient survival, thereby serve as a non-invasive screening tool to detect malignant breast tumor stages. Thus, our findings not only identify a possible mechanism by which breast cancer cells can promote T cell exhaustion and dampen host anti-tumor immunity, but may also identify a target for immune therapy against the most devastating breast tumors.

## Abstract (web summary)

Understanding the factors that hamper immune therapy in breast cancer may increase the range of patients who benefit. Here authors show that breast cancer cells produce and subsequently transfer active TGF-β type II receptors to CD8 + T cells to render them exhausted, thus paralyzing the anti-tumor immune response.

## Introduction

Malignant breast cancers are difficult to cure, especially highly metastatic triple-negative breast cancers (TNBC) lacking estrogen receptor (ER), progesterone receptor (PR) and human epidermal growth factor receptor 2 (HER2) molecules as therapeutic targets^1,2^. Cancer immunotherapies recently emerged as a novel and durable alternative treatment option for certain types of cancer with immune infiltrated phenotype, but for unclear reasons the response rate for breast cancer is low. However, recent clinical trials showed that the immune-checkpoint inhibitors anti-programmed cell death 1 (PD-1) and anti-Programmed cell death 1 ligand 1 (PD-L1) antibodies can promote the response rate in TNBC when combined with chemotherapy or radiotherapy^3,4^, indicating potential benefit for TNBC patients.

One of targetable signaling pathways involved in malignant cancer progression and immune suppression is the transforming growth factor-β (TGF-β) pathway. In TNBC and certain other late-stage tumors, TGF-β signaling cannot suppress cell proliferation and induces and/or enhances epithelial-to-mesenchymal transition (EMT), a cellular program that promotes cancer cell intravasation, immune evasion^5^ and confers cancer stem cells traits associated with high-grade malignancy^6–9^. In addition, TGF-β directly suppresses immune surveillance and protects malignant cells from immune-mediated clearance^10–12^, and stromal TGF-β activity was recently identified as an independent determinant of tumor responsiveness to anti-PD-L1 treatment^13,14^. TGF-β ligands activate cell membrane associated TGF-β type II and type I Ser/Thr kinase receptors (TβRI and TβRII, respectively), which subsequently phosphorylate SMAD2 and 3 to form hetero-oligomer with SMAD4 thus activate transcription^15^. A series of clinical trials aimed at reducing excessive levels of TGF-β ligands or suppressing TGF-β receptor activities have been conducted, yet with limited effects^16^.

Extracellular vesicles (EV), such as endosome-origin “exosomes” and plasma membrane-derived “ectosomes” (microparticles/microvesicles) are defined as the common but functionally specific particles characterized by a wide range of sizes and naturally released from various types of cells^17,18^. EVs contain functional biomolecules (proteins, lipids, RNA and DNA) that can be horizontally transferred to recipient cells^19–23^. Tumor-derived extracellular vesicles (TEV) are emerging regulators of metastasis^24,25^ and the tumor microenvironment^26–30^. Multiple TGF-β central components, including ligand and receptor, were recently identified in EVs of different origin^31–37^. Exosomal TGF-β released by cancer cells can induce fibroblast differentiation into cancer-associated fibroblasts (CAF) that favor invasive growth and metastasis^38,39^. However, whether TEVs containing TGF-β central components could influence T cell exhaustion in the tumor environment is largely unknown.

Here we show that TEVs can transfer activated TβRII to immune cells and pre-malignant tumor cells, thereby triggering host cell SMAD activation and resulting in defective anti-tumor immunity and increased metastasis. High levels of TβRII specifically mark circulating extracellular vesicles (crEV) of malignant breast tumors. These results identify a mechanism by which breast tumor cells can immunocompromise immunity and provide a therapeutic method to improve breast cancer immunotherapy.

## Results

### TGF-β induces extrafacial TβRII on breast tumor cell-derived extracellular vesicles

**Fig. 1:**
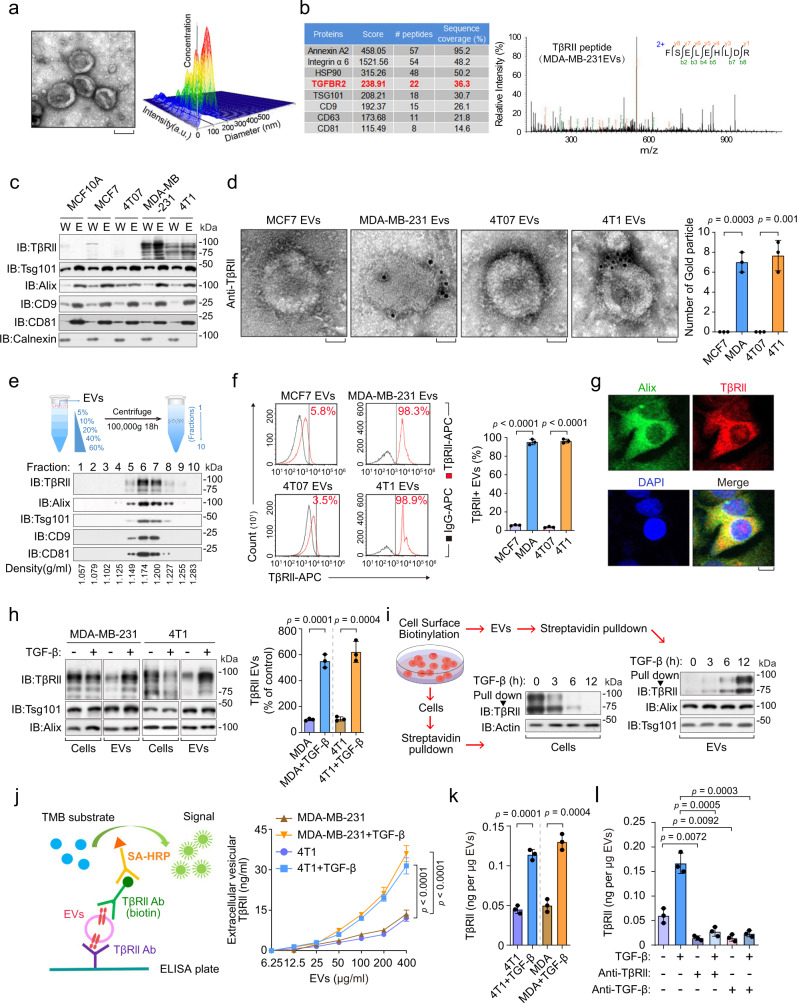
Extrafacial expression of TβRII on malignant breast cancer cell-derived EVs and its regulation by TGF-β. **a** A representative TEM image of purified EVs from MDA-MB-231 cells (left), and nanoparticle tracking of purified EVs (right). Scale bar (left panel), 100 nm. **b** Mass-spectrometry analysis of purified EVs secreted by MDA-MB-231 cells, showing results for Annexin A2, Integrin α6, HSP90, TβRII (TGFBR2, red), TSG101, CD9, CD63 and CD81 (left). TβRII peptide identified by mass-spectrometry analysis of purified EVs from MDA-MB-231 (right panel). **c** Immunoblot detection of TβRII in purified EVs (E) and whole lysates (W) from different breast cancer cell lines. **d** TEM images of different breast cancer cell lines-derived EVs immunogold-labeled with anti-TβRII antibodies (left panel), and quantification of number of gold particles by TEM (right panel). Gold particles are depicted as black dots. Scale bar, 50 nm. **e** Density gradient centrifugation confirming that TβRII secreted by MDA-MB-231 cells co-fractionated with exosome markers Alix and TSG101. **f** FACS analysis and quantification of the percentage of TβRII positive (TβRII^+^) EVs from different breast cancer cell lines (left panel). *n* = 3 biological replicates per group (right panel). The percentage was referred to as the percentage of beads with TβRII^+^ EVs. **g** Co-localization of endogenous TβRII and Alix in MDA-MB-231 cells. Scale bars, 10 μm. **h** Immunoblot analysis (left) and quantification (right) of TβRII in whole cells lysate and EVs derived from control cells and TGF-β-treated cells. **i** Schematic diagram of biotin-labeling assay (left) and immunoblot analysis (right) measuring TβRII levels on the cell membrane and secreted EVs. **j** Schematic diagram (left) of ELISA to measure TβRII concentration (right) on the surface of EVs derived from 4T1 and MDA-MB-231 cells, with or without TGF-β treatment. TMB, 3,3’,5,5’-tetramethylbenzidine; SA-HRP, streptavidin-horseradish peroxidase. **k** ELISA of TβRII on the surface of EVs from indicated cell types. **l**, ELISA of TβRII on EVs isolated from MDA-MB-231 cells pre-treated with or without TGF-β (2.5 ng/ml), TβRII neutralizing antibody (10 µg/ml) or TGF-β neutralizing antibody (10 µg/ml) for 24 h as indicated. *p < 0.05 (two-tailed Student’s t test **d**, **f**, **h**, **k**, **l** or two-way ANOVA **j**). Data are analyzed of three independent experiments and shown as mean ± SD (**d**, **f**, **h**, **j**, **k** and **l**). Source data are provided as a Source Data file.

To compare extracellular vesicles composition of cells representing different stages of breast cancer, we purified EVs from non-tumorigenic (MCF10A), non/low-metastatic (MCF7 and 4T07), and high-metastatic (MDA-MB-231 and 4T1) breast cancer cells by ultracentrifugation, and subsequent transmission electron microscopy (TEM) and NanoSight nanoparticle tracking analysis (Fig. 1a). Ultra-performance liquid chromatography–mass spectrometry (UPLC-MS) revealed abundant and specific presence of TβRII, but not other TGF-β central components such as the TGF-β ligand or SMADs, in extracellular vesicles of the high-metastatic cell types (Fig. 1b and Supplementary Fig. 1a, b). TβRII is rather abundant and specifically enriched in EVs from metastatic breast cancer cells as its expression intensity even exceeds EV markers such as CD9 and CD81 (Fig. 1b). Immunoblotting and immunogold transmission electron microscopy (IG-TEM) also detected TβRII exclusively in extracellular vesicles from metastatic cells and many of the TβRII molecules in the TEVs were found to be anchored to the membrane (Fig. 1c, d). Sucrose density gradient centrifugation confirmed the presence of TβRII in the fraction of EVs (Fig. 1e). Flow cytometry showed that more than 90% of the MDA-MB-231 and 4T1 EVs were positive for TβRII (Fig. 1f and Supplementary Fig. 1c).

Extracellular vesicles are generated and released via a defined intracellular trafficking route^40^. In line with this, immunofluorescence staining of MDA-MB-231 cells showed co-localization of TβRII and the extracellular vesicles marker Alix (Fig. 1g), whereas knockdown of GTPase Rab27A or FYVE finger protein Hrs, which mediates EV secretion, also blocked TβRII secretion via the EVs (Supplementary Fig. 1d, e). Pre-treatment of cells with GW4869, which inhibits the release of extracellular vesicles, also sharply reduced the TβRII levels in the TEVs (Supplementary Fig. 1f). Intriguingly, the level of extracellular vesicle-TβRII (EV-TβRII) secreted by the metastatic breast cancer cells markedly increased upon TGF-β treatment in Hrs-dependent manner, whereas the cellular levels decreased (Fig. 1h and Supplementary Fig. 1g, h), which suggests that after TGF-β stimulation cell surface TβRII is not simply directed for degradation by internalization, but instead secreted into TEVs. To validate this hypothesis, cell surface proteins were labeled with biotin prior to TGF-β treatment. Biotin-labeled TβRII disappeared from the cells and appeared in the EVs after 3-12 h, confirming that the EV-TβRII originates from the MDA-MB-231 cell surface (Fig. 1i). IG-TEM and enzyme-linked immunosorbent assay (ELISA) showed that EV-TβRII had similar membrane topology as cell surface TβRII, with its extracellular domain exposed on the surface of the EVs (Fig. 1d, j). ELISA analysis also confirmed the increased amounts of TβRII in the TEVs upon TGF-β treatment (Fig. 1j, k and Supplementary Fig. 1i), which was abolished by disrupting the TGF-β-TβRII association with neutralizing antibodies (Fig. 1l). Together, these results show the presence of TβRII in the TEVs of malignant breast cancer cells, and increase upon TGF-β treatment.

### TβRII^+^ crEVs are a non-invasive biomarker for metastatic breast cancer

**Fig. 2:**
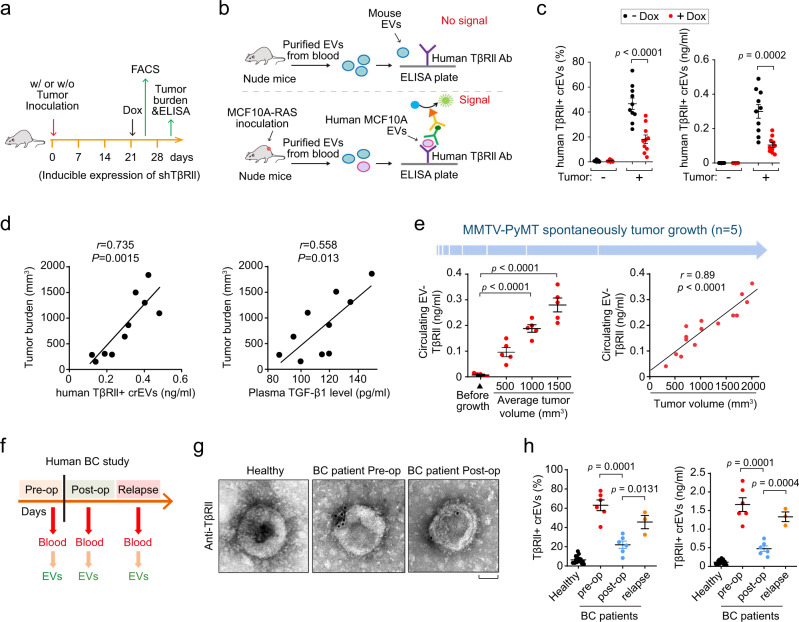
The amounts of TβRII^+^ crEVs positively correlate with tumor burden. **a** Experimental analysis in vivo: mice were not inoculated (−) or were given subcutaneous inoculation (+) of MCF10A-RAS cells (5 × 10^5^ cells per mouse) expressing control shRNA or doxycycline-inducible shRNA targeting TβRII (shTβRII) and tumors were grown for 3 weeks, followed by the administration of doxycycline (Dox). 3 d later, mice were euthanized for further analysis (*n* = 10 mice per group). **b** Schematic of ELISA of human TβRII on EVs in plasma samples from mice with human breast cancer xenograft. **c** FACS analysis (left) of the percentage of TβRII^+^ crEVs and ELISA (right) of TβRII on crEVs in plasma samples from mice (*n* = 10 mice per group). **d** Pearson correlation between the TβRII^+^ crEVs or TGF-β1 in plasma and the tumor burden from the control group mice without Dox administration (*n* = 10 mice per group). **e** Quantification of circulating EV-TβRII at the indicated tumor volume (left panel). Pearson correlation between the circulating EV-TβRII in plasma and tumor burden from mice was shown right (*n* = 5 mice per group). **f** Schematic diagram of longitudinal blood collection pre-operatively, post-operatively or relapse. **g** Electron microscopy images of EVs secreted from healthy donors, patients with pre- and post-operatively breast cancer. Scale bars, 50 nm. **h** FACS (left) and ELISA (right) of TβRII^+^ crEVs in plasma samples from patients with breast cancer pre-operation (*n* = 6 patients), post-operation (*n* = 6 patients), relapse (*n* = 3 patients) and healthy donors (*n* = 20 samples). *p < 0.05 (two-tailed Student’s t test (**c**, **e** (left), **h**) or two-way ANOVA (**d**, **e** (right))). Data are analyzed of three independent experiments and shown as mean ± SD (**c**, **e** (left), **h**). Source data are provided as a Source Data file.

To investigate the secretion of EV-TβRII by breast cancer cells in vivo, we established human MCF10A-RAS xenografts carrying doxycycline-inducible TβRII silencing short hairpin RNA (shRNA) (Fig. 2a). Blood from these mice was collected for detecting the percentage of TβRII^+^ crEVs by FACS, or EV-TβRII proteins by ELISA (Fig. 2b). Antibodies against human TβRII identified human TβRII on crEVs from mice bearing human MCF10A-RAS xenografts but not on crEVs from the control mice (Fig. 2b). Doxycycline-mediated transient loss of TβRII sharply reduced the abundance of TβRII^+^ crEVs (Fig. 2c). The level of TβRII^+^ crEVs, as well as plasma TGF-β1 level, positively correlated with tumor burden (Fig. 2d). To further substantiate these results, we examined the well-established murine mammary tumor virus (MMTV)-polyoma middle T antigen (PyMT) transgenic mouse breast cancer model, crEVs were assessed for the presence of TβRII when the PyMT-induced tumors reached an average volume of 500, 1000 and 1500 mm^3^; The circulating EV-TβRII was found to increase proportionally with tumor size and also associated with the levels of metastasis (Fig. 2e and Supplementary Fig. 2a, b). Furthermore, we evaluated TβRII crEVs in plasma of breast cancer patients at pre- and post-surgery stages (post-operative day 7; Fig. 2f). We found that all cases involved in the test with longitudinal blood collections showed a significant decrease in TβRII^+^ crEVs levels after surgical resection (average from 63.06% to 22.05%; average from 1.66 ng/ml to 0.47 ng/ml) and a significant increase of TβRII^+^ crEVs levels in relapsed breast cancer patients (average from 22.05% to 43.45%; average from 0.47 ng/ml to 1.33 ng/ml) (Fig. 2g, h).

**Fig. 3:**
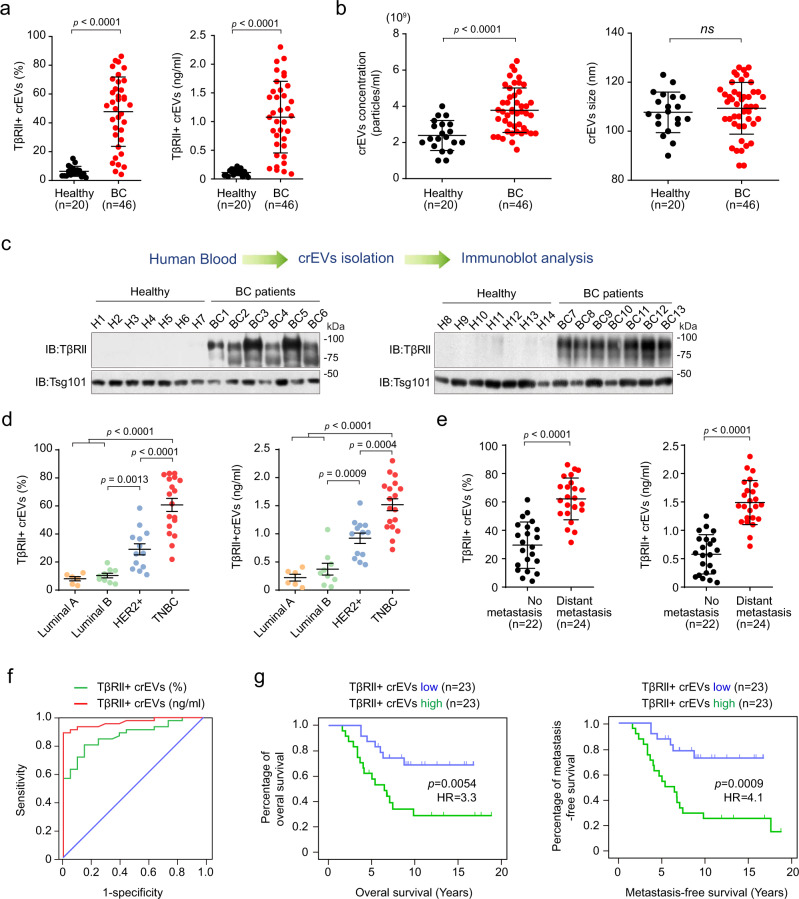
The amount of TβRII on circulating EVs distinguishes patients with breast cancer from healthy donors. **a** FACS analysis (left panel) of the percentage of TβRII^+^ crEVs and ELISA (right panel) of TβRII on crEVs in plasma samples from healthy donors (*n* = 20 samples) and patients with breast cancer (*n* = 46 samples). The number of particles used in each FACS-based assessment is the same (10,000 events per sample). **b** Nanosight analysis of the concentration (left) and size (right) of the TβRII^+^ crEVs in plasma from healthy donors (*n* = 20 samples) and patients with breast cancer (*n* = 46 samples). **c** Immunoblot analysis of crEVs in plasma from healthy donors (*n* = 14 samples, H1-H14) and patients with breast cancer (*n* = 13 samples, BC1-BC13). The protein loading of EVs of healthy and BC samples was equal. **d** FACS (left panel) and ELISA (right panel) of TβRII^+^ crEVs in breast cancer patients of different subtypes (Luminal A, *n* = 6 samples; Luminal B, *n* = 9 samples; HER2^+^, *n* = 13 samples; TNBC, triple-negative breast cancer, *n* = 18 samples). **e** FACS (left) and ELISA (right) of TβRII^+^ crEVs in patients with no metastasis (*n* = 22 samples) and patients with distant metastasis (*n* = 24 samples). **f** ROC curve analysis for the indicated parameters in patients with breast cancer (*n* = 46 samples) compared to healthy donors (*n* = 20 samples). **g** Kaplan-Meier curves (log-rank test) displaying overall (left panel) and metastasis free (right panel) survival of patients with a high (>46%, FACS-detected level) TβRII^+^ crEVs (green), and with a low (<46%, FACS-detected level) TβRII^+^ crEVs (blue). *p < 0.05 (two-tailed Student’s t test **a**, **b**, **d**, **e** or two-way ANOVA **f**, **g**). Data are analyzed of three independent experiments and shown as mean ± SD **a**, **b**, **d**, **e**. Source data are provided as a Source Data file.

Next, we compared crEVs from breast cancer patients (*n* = 46) and healthy donors (*n* = 20). crEVs from healthy donors showed very low baseline positivity for TβRII in, ranging from 1.9 to 15.3% (average 6.3%; fluoresecence activated cell sorting (FACS) analysis), and from 0.03 to 0.22 ng/ml (average 0.11 ng/ml; in ELISA) (Fig. 3a). 89% of breast cancer patients (41 out of 46) showed much higher percentage and amount of the TβRII ^+^ crEVs (*P* < 0.0001; Fig. 3a and Supplementary Table 1, 2). In the sera of the cancer patients the amount of crEVs was significantly higher than in the healthy donors, but the average size was comparable (Fig. 3b). Immunoblotting confirmed the strong and specific expression of TβRII in the crEVs of breast cancer patients (Fig. 3c). Patients with triple-negative breast cancer showed significant higher percentage and amount of TβRII in crEVs than that of HER2^+^ patients, and both were much higher than those of luminal patients (Fig. 3d). Moreover, patients with distant metastasis showed significantly higher levels of TβRII in crEVs (average 62.19% or 1.49 ng/ml) than patients without metastasis (average 29.58% or 0.58 ng/ml; Fig. 3e). Considering that EV-TβRII increased proportionally with tumor size and metastasis in mice models (Fig. 2d, e and Supplementary Fig. 2a, b), the increased levels of EV-TβRII in patients with metastasis might also represent increased tumor burden. Notably, when comparing breast cancer patients to healthy donors, the receiver operating characteristic (ROC) curves showed that the percentage of TβRII^+^ crEVs could be a nice classifier with an area under curve (AUC) of 0.872 exhibiting a sensitivity of 93.48% and a specificity of 90%, an accuracy of 92.42%, and with a positive predictive value of 95.55% and negative predictive value of 85.71% (Fig. 3f and Supplementary Table 3). The TβRII ELISA gave similar results to FACS assay: ROC curves indicated that circulating EV-TβRII protein shows an AUC of 0.967 a sensitivity of 89.13%, a specificity of 100%, an accuracy of 92.42%, and positive and negative predictor values of 100% and 80%, respectively (Fig. 3f and Supplementary Table 3), supporting its utility as a biomarker for breast cancer and its potential for early detection of metastasis. To determine the prognostic relevance of TβRII^+^ crEVs in breast cancer, patients were dichotomized into high- versus low- groups on the basis of a median split of TβRII^+^ crEVs value. Apparently, patients with a lower level of TβRII^+^ crEVs showed improved overall survival and metastasis-free survival when compared to those exhibiting higher levels of TβRII^+^ crEVs (Fig. 3g). Using similar cut-off strategy, ELISA for circulating TβRII could function with similar specificity and sensitivity as TβRII^+^ crEVs analysis (Supplementary Fig. 2c), consolidating that circulating TβRII is a non-invasive biomarker for metastatic breast cancer.

### EV-TβRII mediates TEVs-induced SMAD activation in recipient cells that promotes metastatic outgrowth in tumors

**Fig. 4:**
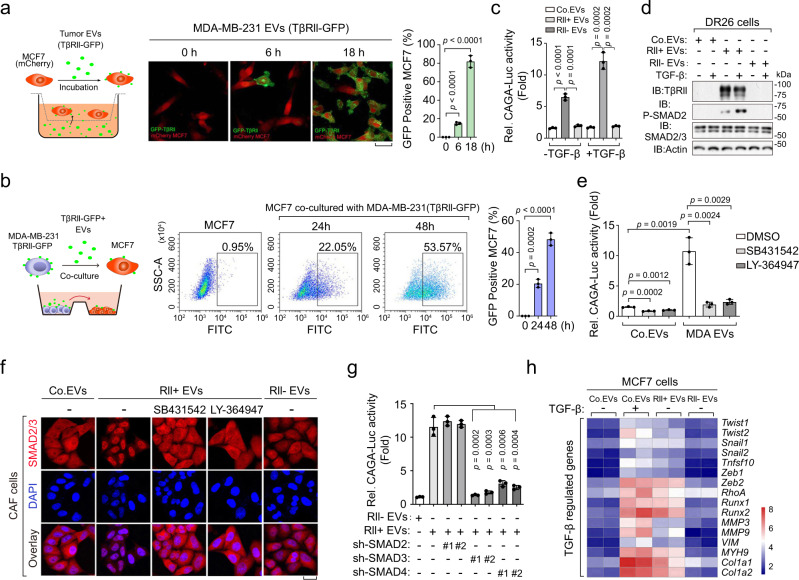
EV-TβRII mediates the TEVs-induced SMAD activation in recipient cells. **a** Schematic diagram (left panel), confocal microscopy (middle panel) and quantification (right panel) of MCF7 cells incubated with EVs derived from MDA-MB-231 cells stably expressing TβRII-GFP for indicated times. Scale bar, 20 μm. **b** Schematic diagram (left panel), FACS analysis (middle panel) and quantification (right panel) of MCF7 cells incubated with MDA-MB-231 cells stably expressing TβRII-GFP for indicated times. **c** Transcriptional analysis of TGF-β-induced (2.5 ng/ml for 16 hrs) CAGA_12_-Luc response in TβRII-deficient mink lung epithelial DR26 cells pre-treated for 24 h with empty liposome (as a control; Co.EVs), TβRII^+^ (RII+) EVs (40 μg) derived from untransfected MDA-MB-231 cells or TβRII^−^(RII−) EVs (40 μg) derived from TβRII knockout MDA-MB-231 cells. **d** Immunoblot analysis of total lysates of DR26 cells pre-incubated with empty liposome (as a control; Co.EVs) or EVs (40 μg) derived from TβRII^+^ (RII+) or TβRII^−^(RII−) MDA-MB-231 cells, and stimulated with TGF-β (2.5 ng/ml) for 1 h as indicated. **e** Transcriptional analysis of CAGA_12_-Luc response in TβRII-deficient DR26 cells incubated with empty liposome (as a control; Co.EVs) or EVs (40 μg) derived from TβRII^+^ (RII+) MDA-MB-231 cells (MDA EVs) and treated with control DMSO, SB431542 (10 μM) or LY-364947 (10 μM) as indicated. **f** Immunofluorescence and DAPI staining of CAF cells pre-incubated with Co.EVs, TβRII^+^ or TβRII^−^ EVs (40 μg) and treated with control DMSO, SB431542 (10 μM) or LY-364947 (10 μM) as indicated. Scale bar, 20 μm. **g** Transcriptional analysis of CAGA_12_-Luc response in HEK293T cells as indicated pre-treated for 24 h with EVs (40 μg) derived from TβRII^+^ (RII+) or TβRII^−^(RII−) MDA-MB-231 cells. **h** qPCR analysis in MCF7 cells pre-incubated for 48 h with Co.EVs, TβRII^+^ or TβRII^−^ EVs (40 μg), followed by no stimulation (−) or stimulation (+) for 16 h with TGF-β (2.5 ng/ml). Relative mRNA levels are shown as a heatmap. *p < 0.05 (two-tailed Student’s t test (**a**, **b**, **c**, **e**, **g**)). Data are analyzed of three independent experiments and shown as mean + SD (**a**, **b**, **c**, **e**, **g**). Source data are provided as a Source Data file.

To visualize transfer of EV-TβRII from donor cells to the recipient cells, we stably expressed TβRII-GFP in MDA-MB-231 and purified the EVs of these cells. Immunofluorescence and FACS analysis showed that TβRII-GFP accumulated both on the cell surface and on the inside of mcherry-labeled MCF7 cells after treatment with these TβRII-GFP containing EVs (Fig. 4a). To address the EV transfer from one cell to another, we have performed further analysis by co-culturing MCF7 with MDA-MB-231 that stably expressing TβRII-GFP. FACS analysis showed that, after co-culture, the TβRII-GFP molecules were found to accumulate on the cell surface of MCF7 cells (Fig. 4b). To investigate whether the administration of TβRII^+^ TEVs can cause TGF-β/SMAD activation, we deleted the TβRII encoding gene in MDA-MB-231 cells by CRISPR-Cas9 technology. Immunoblot analysis confirmed the absence of TβRII in TEVs from cells in which the TβRII-encoding gene was deleted (Supplementary Fig. 3a). Pre-treatment of TβRII-deficient mink lung epithelial DR26 cells^41^ with TβRII^+^ MDA-MB-231 EVs (purified from non-transfected MDA-MB-231 cells) sharply increased SMAD3/SMAD4-dependent CAGA-luc transcriptional reporter activity; It thus substantially restored the responsiveness of DR-26 cells to TGF-β ligand, and established both basal and TGF-β-induced SMAD2 phosphorylation, whereas pre-treatment with TβRII^−^ EVs (purified from TβRII-deficient MDA-MB-231 cells) did not show this (Fig. 4c, d). These results indicate that TβRII transferred by TEVs induces TGF-β/SMAD activity.

Interestingly, pre-treatment of TβRII^+^ EVs receiving cells with the TβRI kinase inhibitors SB431542 and LY-364947 blocked TEVs-induced CAGA-luc activity (Fig. 4e). Similar results were obtained for cancer-associated fibroblasts (CAF), in which TEVs-induced SMAD2/3 nuclear translocation could be blocked by pre-treatment with TβRI kinase inhibitors (Fig. 4f). We also found that, knocking down of SMAD3/4, but not SMAD2, in recipient cells could abolish TβRII^+^ EVs induced CAGA-luc transcriptional reporter activity (Fig. 4g and Supplementary Fig. 3b), suggesting that EV-TβRII activate recipient SMAD3/SMAD4 signaling cascade. Incubation of MCF7 cells with TβRII^+^ EVs up-regulated TGF-β-SMAD3/4 target genes to a level similar as with TGF-β treatment, including key transcriptional inducers of EMT and markers of cancer stemness (Fig. 4h and Supplementary Fig. 3c, d). Treatment with TGF-β stimulates TβRII internalization and incorporation into EVs. To explore the effect of EV-TβRII in recipient cells, we examined the ubiquitination status of TβRII in EVs from TβRII-Flag expressing cells pre-treated with or without TGF-β. Ubiquitination is clear on TβRII from EVs and there is no significant difference between the control and TGF-β treated samples (Supplementary Fig. 3e). Using antibodies specific for K48- or K63- poly-Ub chains, we observed that mainly the K63-chains, rather than K48-chains, were conjugated to EV-TβRII (Supplementary Fig. 3e). This result indicated that EV-TβRII received in recipient cells may not necessarily translated into degradation and play significant roles in recipient cells.

**Fig. 5:**
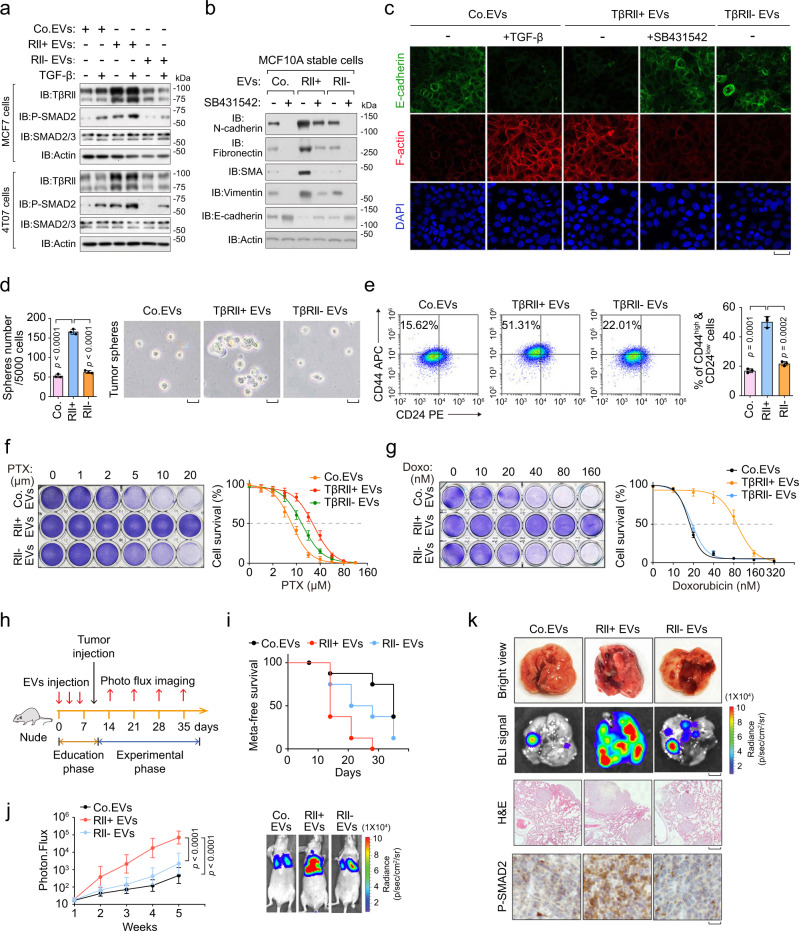
EV-TβRII promotes breast cancer stemness and metastatic outgrowth. **a** Immunoblot analysis of total cells lysates from MCF7 cells (upper panel) or 4T07 cells (lower panel) pre-treated for 24 h with TβRII^+^ EVs or TβRII^−^ EVs (40 μg) derived from MDA-MB-231 cells, and stimulated with or without TGF-β (5 ng/ml) for 1 h as indicated. **b** Immunoblot analysis of total cell lysate of MCF10A-RAS cells pre-treated for 48 h with TβRII^+^ (RII+) EVs or TβRII^−^ (RII−) EVs (40 μg) derived from MDA-MB-231 cells, and treated with or without SB431542 (10 μM) for 48 h as indicated. **c** Immunofluorescence and DAPI staining of HaCaT cells pre-treated for 48 h with TβRII^+^ (RII+) EVs or TβRII^−^ (RII−) EVs (40 μg) derived from MDA-MB-231 cells, and treated with or without TGF-β (5 ng/ml) and SB431542 (10 μM) for 48 h as indicated. Scale bars, 20 μm. **d** MCF10A-RAS cells pre-treated with control EVs, TβRII^+^ (RII+) EVs, or TβRII^−^ (RII−) EVs (40 μg) derived from MDA-MB-231 cells were analyzed in a tumor sphere assay. Quantification of the number of tumor spheres per 5 × 10^3^ seeded cells (left panel) and pictures show representative images of tumor spheres (right panel). Scale bar, 2 mm. **e** FACS analysis (left panel) and Quantification (right panel) of the CD44^high^/CD24^low^ population in MCF10A-RAS cells treated with Co.EVs, TβRII^+^ or TβRII^−^ EVs (40 μg) for 48 h. **f** Cytotoxic crystal violet staining (left) and dose-response curves (right) to paclitaxel (PTX) of MDA-MB-231 cells pre-incubated with Co.EVs, TβRII^+^ or TβRII^−^ EVs (40 μg) for 48 h. The dose is presented on the x axis, while cell viability (% vs control) is presented on the y axis. **g** Cytotoxic crystal violet staining (left) and dose-response curves (right) to Doxorubicin (Doxo) of MDA-MB-231 cells pre-incubated with Co.EVs, TβRII^+^ or TβRII^−^ EVs (40 μg) for 48 h. The dose is presented on the x axis, while cell viability (% vs control) is presented on the y axis. **h**–**k** EVs administration and experimental analysis in vivo: 4T07 cells pre-treated with Co.EVs, TβRII^+^ or TβRII^−^ EVs (40 μg) were tail vein-injected into nude mice (*n* = 8 mice per group). Mice were also given injection (into the tail vein) of corresponding EVs (50 μg per mouse every other day) before the tumor injection **h**; the percentage of metastasis-free mice in each experimental group followed in time **i**; Lung metastasis was measured by BLI. Normalized photon flux (left panel), representative images (right panel) in each experimental group followed in time **j**; Representative images of bright view (upper), BLI signal (scale bars, 2 mm, middle upper), HE (scale bars, 1 mm, middle lower) and IHC staining (scale bars, 2 μm, lower) in each group **k**. *ns* not significant (*p* > 0.05) and *p < 0.05 (unpaired two-tailed Student’s t test **d**, **e** or two-way ANOVA **f**, **g**, **j**). Data are analyzed from three independent experiments and shown as mean ± SD **d**, **e**, **f**, **g**, **j**. Source data are provided as a Source Data file.

Moreover, in MCF-7 and pre-malignant 4T07 breast cancer cells treated with TβRII^+^ EVs, we detected higher levels of TβRII and downstream phosphorylated-SMAD2 (P-SMAD2) than in cells treated with TβRII^−^ EVs (Fig. 5a). In line with above results, TβRII^+^ EVs-treated epithelial cells exhibited a mesenchymal-like phenotype similar as induced by TGF-β (Fig. 5b, c). As EMT contributes to metastasis and is intimately wired to tumor-initiating capacity^42^, we examined whether TEVs could induce self-renewal potential and stemness as assayed by mammosphere formation and propagation. Indeed, the number of tumor-like mammospheres of MCF10A-RAS cells in suspension culture sharply increased upon pre-treatment with TβRII^+^ EVs (Fig. 5d). In addition, TβRII^+^ EVs, but not TβRII^−^ EVs, induced *en masse* enlargement of the CD44^high^/CD24^low^ population (Fig. 5e), which marks an increase in cancer stem cells (CSC). In line with the fact that cancer-initiating cells often show increased resistance to chemotherapy^43–45^, MDA-MB-231 cells pre-treated with TβRII^+^ EVs were more resistant to Paclitaxel (PTX) and Doxorubicin (Doxo) (Fig. 5f, g). These results suggested that TβRII^+^ EVs exhibit a critical role in cancer stemness, metastasis, and chemotherapy resistance.

To determine whether TβRII^+^ EVs regulate metastatic outgrowth of breast cancer cells in vivo, we used tail vein injection of 4T07 cells in nude mice and ‘educated’ the mice with MDA-MB-231 cells-derived EVs (Fig. 5h). Education with TβRII^+^ EVs resulted in significantly increased lung metastasis and reduced metastatic-free survival, whereas education with TβRII^−^ EVs had much weaker effect (Fig. 5i–k and Supplementary Fig. 4a, b). The numbers of circulating tumor cells (CTC), a hallmark of metastasis, were severely increased in the group educated with TβRII^+^ EVs (Supplementary Fig. 4c). Immunohistochemistry showed an increase of the TβRII and phosphorylated (P)-SMAD2 levels in the metastatic lesions from TβRII^+^ EVs-educated mice (Fig. 5k and Supplementary Fig. 4d). Together, these analyses show that EV-TβRII strongly promotes metastatic outgrowth.

### EV-TβRII antagonizes the anti-tumor immunity in vivo

**Fig. 6:**
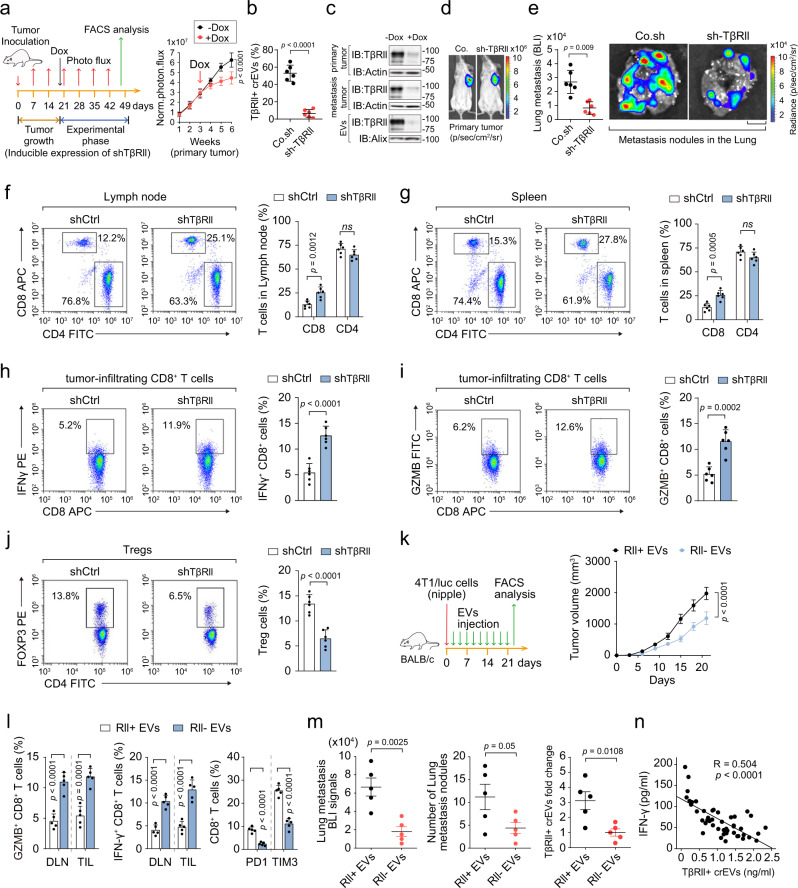
EV-TβRII antagonizes the anti-tumor immunity and thus elevates tumor growth. **a**–**j** Experimental analysis in vivo: BALB/c mice were nipple injectied with 4T1 cells (2 × 10^5^ cells per mouse) expressing control shRNA or doxycycline-inducible shRNA targeting TβRII (shTβRII) and tumors were grown for 3 weeks, followed by the administration of doxycycline (Dox) (**a** left panel). Primary tumor formation in each group at day 21 after implantation is shown (*n* = 6 mice per group); Normalized BLI signals (**a** right panel). Percentage of TβRII^+^ crEVs in plasma from mice **b**. Immunoblot analysis of TβRII in primary tumor, metastatic tumor and circulating EVs from plasma in mice **c**. Representative BLI signals of primary tumor **d**. Normalized BLI signals (left panel) and representative bioluminescent view of isolated lung (right panel) **e**; Scale bar, 2 mm. FACS analysis and quantification of the percentage of CD8^+^ or CD4^+^ cells from lymph nodes **f** and spleen **g**; FACS analysis and quantification of IFNγ^+^ **h** or GZMB^+^ **i** of CD8^+^ cells in tumor-infiltrating lymphocytes (TIL) populations from mice. FACS analysis and quantification of the percentage of tregs **j**. **k** Experimental analysis in vivo: BALB/c mice were nipple injected with 4T1 cells (5 × 10^5^ cells per mouse), followed by tail vein-injection of EVs derived from control 4T1 cells (TβRII^+^) or TβRII knock-out 4T1 cell (TβRII^−^) (50 μg per mouse every other day) for 3 weeks (left) (*n* = 5 mice per group). Tumor volume measured in time (right). **l** Quantification of the percentage of GZMB^+^ of CD8^+^ cells (left panel), IFNγ^+^ of CD8^+^ cells (middle panel) from draining lymph node (DLN) and tumor-infiltrating lymphocyte (TIL) and PD1^+^, TIM3^+^ of CD8^+^ cells from TIL (right panel). **m** BLI signals of lung metastasis (left panel), number of lung metastasis nodules (middle panel) and percentage of TβRII^+^ crEVs in plasma (right panel) of all mice in each experimental group at week 5 were shown. **n** Pearson correlation analysis of the ELISA-detected levels of IFN-γ and TβRII^+^ crEVs in patients with breast cancer (*n* = 46 samples). *ns*, not significant (*p* > 0.05) and *p < 0.05 (unpaired two-tailed Student’s t test **b**, **e**, **f**–**j**, **l**, **m** or two-way ANOVA **a**, **k**, **n**). Data are analyzed from three independent experiments and shown as mean + SD **f**–**j**, **l** or as means ± SD **a**, **b**, **e**, **k**, **m**. Source data are provided as a Source Data file.

To validate the role of EV-TβRII in vivo, we injected 4T1 cells containing a doxycycline-inducible TβRII shRNA vector under the nipple of BALB/c mice and treated the mice with doxycycline 3 weeks after inoculation (Fig. 6a). The level of TβRII^+^ crEVs in the plasma of mice carrying a control shRNA ranged from 40% to 68% (average, 52%; Fig. 6b). Doxycycline-induced loss of TβRII for 3 days did not significantly alter tumor volume but sharply reduced the abundance of TβRII^+^ crEVs, (Fig. 6b, c). After 4 weeks of continuous Dox treatment, tumors under the nipple started to showed signs of slower growth while lung metastases were greatly reduced (Fig. 6d, e and Supplementary Fig. 5a). Immunophenotyping of the spleen and lymph node showed increased percentages of CD8^+^ T cells after doxycycline-induced TβRII depletion in the tumors (Fig. 6f, g and Supplementary Fig. 5b). We also observed increases in the percentages of tumor-infiltrating lymphocytes (TIL) producing IFNγ and activation marker Granzyme B (GZMB), and a reduced fraction of FOXP3^+^ T regulatory cells (Fig. 6h–j and Supplementary Fig. 5b), whereas the fraction of CD11b^+^ Ly6G^+^ neutrophils and F4/80^+^ CD11b^+^ macrophages remained constant (Supplementary Fig. 5c).

To investigate whether exogenously introduced EV-TβRII could suppress the anti-tumor immune response and promote tumor growth, we transplanted 4T1 cells in mice followed by tail vein injections of in vitro collected TEVs from either their WT or TβRII null counterparts (Fig. 6k). Injection of EVs from the WT, but not TβRII null, cells more strongly promoted tumor growth (Fig. 6k). EVs from the WT cells, but not TβRII null, led to a reduced cytotoxicity of tumor-infiltrating T cells (IFNγ^+^ or GZMB^+^ of CD8^+^ cells) and an increased exhaustion of tumor-infiltrating T cells (IFNγ^+^, PD1^+^ or TIM3^+^ of CD8^+^ cells) (Fig. 6l). In line with the impaired anti-tumor immunity, the levels of cancer metastases as measured from the signals and number of lung metastatic nodules, were strongly elevated by EVs from the WT cells, but not TβRII null counterparts (Fig. 6m).

To investigate whether EV-TβRII could rescues the tumor growth rate in the absence of TβRII, we transplanted 4T1 cells expressing a doxycycline (DOX)-inducible shRNA targeting TβRII in mice followed by DOX administration and tail vein injection of in vitro collected TEVs from either their WT or TβRII null counterparts (Supplementary Fig. 6a). The TβRII expressions in primary and metastatic tumors were also elevated after TβRII^+^ EVs treatment (Supplementary Fig. 6b, c). As shown, in doxycycline-treated mice, education with TβRII containing EVs can partially rescue the tumor growth rate and lung metastasis (Supplementary Fig. 6a, d, e). We also observed that TβRII^+^ EVs could led to a reduced cytotoxicity of tumor-infiltrating T cells (IFNγ^+^ or GZMB^+^ of CD8^+^ cells) and an increased exhaustion of tumor-infiltrating T cells (IFNγ^+^ or PD1^+^ of CD8^+^ cells) and a reduced fraction of FOXP3^+^ T regulatory cells in doxycycline-treated mice (Supplementary Fig. 6f).

To evaluate how CD8^+^ T cells affect the pro-tumorigenic/pro-metastatic function of TβRII^+^ TEVs, we also transplanted 4T1 cells to control and the CD8^+^ T cell-depleted host (Supplementary Fig. 7a). Antibody-based depletion of CD8^+^ cells significantly promoted tumor growth and metastasis; Mice educated with TβRII^+^ EVs also showed significantly increased tumor burden (Supplementary Fig. 7b–e). Combined CD8^+^ T cells depletion with TβRII^+^ EVs education together could show addictive or synergistic tumor-promoting effects (Supplementary Fig. 7c–e), indicating that TβRII^+^ EVs education might promote tumor progression and metastasis through simultaneously inducing a more aggressive phenotype in tumor cells and immune suppression in host. Moreover, the abundance of TβRII^+^ crEVs inversely correlated with interferon (IFN)-γ production (Fig. 6n), suggesting that the secretion of EV-TβRII from tumor cells might negatively affect anti-tumor immunity of the host. Immunohistochemical analysis on some previously collected and preserved patient samples also showed that the level of TβRII^+^ crEVs in plasma were found to be negatively correlated with the level of tumor-infiltrating CD8^+^ T cells (Supplementary Fig. 7f). Together, these results revealed that EV-TβRII inhibits anti-tumor immunity in mice.

### EV-TβRII induces exhaustion of CD8^+^ T cells

**Fig. 7:**
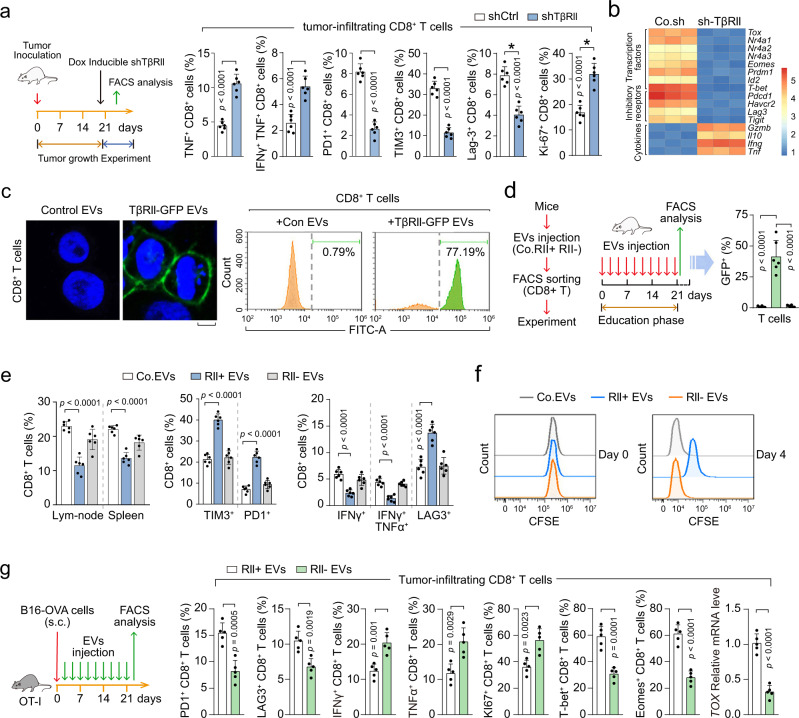
EV-TβRII mediates the exhaustion of CD8^+^ T cells by TEVs. **a** Experimental analysis in vivo: BALB/c mice were nipple injectied with 4T1 cells (2 × 10^5^ cells per mouse) expressing control shRNA or doxycycline-inducible shRNA targeting TβRII (shTβRII) and tumors were grown for 3 weeks (*n* = 6 mice per group), followed by the administration of doxycycline (Dox) as in Fig. 6a (left panel). Frequency of CD8^+^ T cells expressing TNFα, IFNγ, PD1, TIM3, LAG-3, and Ki-67 in tumor-infiltrating lymphocytes (TIL) populations from mice (right panel). **b** qRT-PCR analysis of CD8 T cells in TILs from mice and the results shown as a heatmap. **c** Confocal microscopy (left panel) and FACS analysis (right panel) of purified CD8^+^ T cells pre-treated with control EVs or EVs (40 μg) derived from MDA-MB-231 cells expressing TβRII-GFP for 48 h. Scale bar, 5 μm. **d**–**f** Experimental analysis in vivo: BALB/c mice were tail vein-injected of control EVs, TβRII-GFP^+^ EVs, or TβRII^−^ EVs for 3 weeks (*n* = 6 mice per group); then the percentage of GFP positive CD8^+^ T cells of mice blood were quantified **d**. The percentage of CD8^+^ T cells in lym-node or spllen and TIM3^+^, PD1^+^, IFNγ^+^, IFNγ^+^&TNFα^+^, LAG3^+^ of CD8^+^ T cells in TIL were analyzed by FACS (*n* = 6 mice per group) **e**. The cellular proliferation of CD8^+^ T cells were analyzed by CFSE dilution **f**. **g** Experimental analysis in vivo: OT-I TCR transgenic mice were subcutaneous injected with B16 melanoma cells expressing MHC class I specific epitope of OVA (B16-OVA), followed by tail vein-injection of EVs derived from control (TβRII^+^) or TβRII knock-out (TβRII^−^) 4T1 cell (50 μg per mouse every other day) for 3 weeks (*n* = 5 mice per group) (left); Quantification of the percentage of PD1^+^, LAG3^+^, IFNγ^+^, TNFα^+^, Ki67^+^, T-bet^+^, Eomes^+^ of CD8^+^ T cells and qPCR analysis of *TOX* mRNA from tumor-infiltrating lymphocyte (TIL). *p < 0.05 (two-tailed Student’s t-test **a**, **d**, **e**, **g**). Data are analyzed of three independent experiments and shown as mean + SD **a**, **d**, **e**, **g**. Source data are provided as a Source Data file.

Anti-tumor immunity is hindered by T-cell expression of inhibitory molecules associated with an exhausted phenotype. T cell exhaustion is characterized by high and sustained expression of inhibitory receptors, progressive loss of capacity of producing effector cytokines, diminished proliferating capacity, and distinct transcriptional and epigenetic programs^46^. We found that TβRII depletion in the tumors induced much higher frequencies of 4T1-specific CD8^+^ T cells secreting effector cytokines such as GZMB, IFNγ and TNFα (Fig. 7a). Moreover, we observed higher levels of inhibitory receptors such as PD1, TIM3, LAG-3, and a decreased proliferating capacity of 4T1-specific CD8^+^ T cells at the tumor site in control mice (Fig. 7a). Furthermore, TβRII^+^ EVs induced transcriptome-wise changes in CD8^+^ T cells also showed an increase of transcription factors and inhibitory receptors and a decrease of cytokines, which reflects the T cell exhaustion (Fig. 7b). These results suggested that tumor cell-derived EV-TβRII indeed may drive T cell exhaustion thus suppress anti-tumor immunity. To examine whether the administration of TβRII^+^ TEVs would be sufficient to transfer to CD8^+^ T cells, we purified CD8^+^ T cells from mice and incubated them with TβRII-GFP^+^ EVs. 80% of the purified cells acquired GFP signals (Fig. 7c). Together, these results show that TβRII released from TEVs induce exhaustion of CD8^+^ T cells.

To determine whether TβRII^+^ TEVs regulate anti-tumor immunity of the host in vivo, we injected 4T1 cell-derived TEVs into the tail vein of mice and performed FACS analysis after 3 weeks. In the group educated with TβRII-GFP^+^ 4T1 EVs, close to 50% of the CD8^+^ T cells were GFP positive (Fig. 7d), indicating that TEVs can transfer TβRII-GFP to these immune cells with high efficiency in vivo. In line with this, significantly lower percentages of CD8^+^ T cells, decreased cytokines production by CD8^+^ T cells, increased expression of inhibitory receptors such as PD1, TIM3, or LAG-3 on CD8^+^ T cells, and the suppressed cellular proliferation of CD8^+^ T cells, were found in spleens and lymph nodes from mice educated with TβRII-GFP^+^ TEVs, but not in mice educated with TβRII^−^ TEVs (Fig. 7e, f).

To examine the phenotype and function of tumor antigen-specific CD8^+^ T cells by TβRII^+^ EVs, we injected OT-I TCR transgenic mice with B16 melanoma cells expressing MHC class I specific epitope of OVA (B16-OVA) (Fig. 7g). Compared with TβRII^−^ EVs, education with TβRII^+^ EVs led to significantly increased levels of inhibitory immune checkpoint receptors (PD1, LAG-3), decreased cytokine production (IFN-γ, TNF-α) and reduced proliferating capacity (Ki-67) of OVA peptide-specific CD8^+^ T cells, resulting in T-cell exhaustion (Fig. 7g). Exhausted CD8^+^ TILs include a subpopulation of progenitor exhausted cells that retain polyfunctionality^47^, persist long term and differentiate into terminally exhausted TILs. It is known that TCF-1 is necessary to support this progenitor-like exhausted cells^48,49^, and the T-box transcription factors T-bet and Eomes are associated with terminally exhausted T cells^47,49^. We examined the expression of T-bet and Eomes of the tumor-specific CD8 T cells by FACS. Compared with TβRII^−^ EVs, TβRII^+^ EVs induced exhausted CD8^+^ T cells had increased T-bet and Eomes levels, indicating that TβRII^+^ EVs induced exhausted CD8^+^ T cells are terminally exhausted cells. TOX has been reported to be a key DNA-binding factor for T-cell exhaustion^50–54^. Compared with TβRII^−^ EVs group, *TOX* mRNA level was up-regulated in OVA-specific CD8^+^ T cells in control mice treated with TβRII^+^ EVs group (Fig. 7g), indicating that TβRII^+^ EVs induced exhaustion of CD8^+^ T cell involves the expression of TOX. These results confirm that EVs of breast tumor are functioning through TβRII to boost T cell exhaustion and thereby promote tumor growth.

### TCF1 partners (cooperates) with activated SMAD3 to impose CD8^+^ T cell exhaustion

**Fig. 8:**
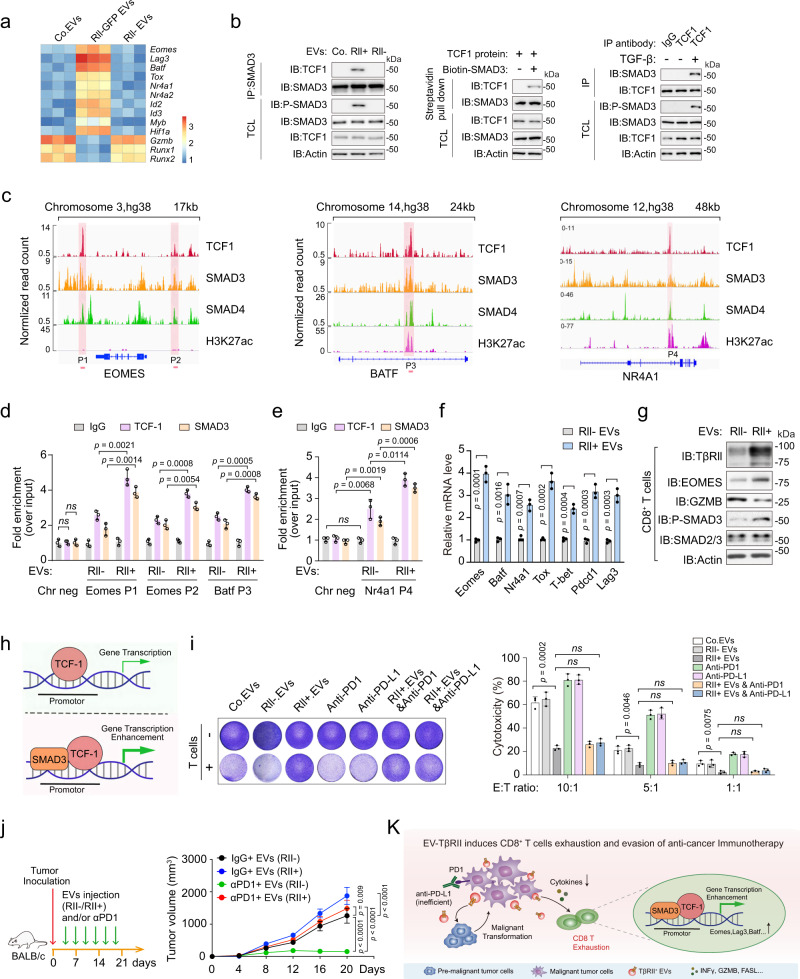
TCF1 partners (cooperates) with SMAD3 to impose CD8^+^ T cell exhaustion. **a** qRT-PCR analysis of CD8^+^ T cells from mice in Fig. 7g and the results shown as a heatmap. **b** Immunoblot (IB) analysis of anti-SMAD3 immunoprecipitates from purified CD8^+^ T cells pre-incubated with Co.EVs, TβRII^+^ (RII+) or TβRII^−^ (RII−) (40 μg) for 48 h (left panel); Purified TCF1 and SMAD3 interaction in vitro: Eukaryotic purified TCF1 proteins and prokaryotic purified SMAD3 were incubated and immunoprecipitated with streptavidin beads (middle panel); IB of anti-TCF1 immunoprecipitate derived from purified CD8^+^ T cells treated with or without TGF-β (5 ng/ml) as indicated (right panel). **c** Genome Browser tracks from ENCODE in HepG2 cells representing the binding sites of TCF1, SMAD3, SMAD4 and H3K27ac at the *Eomes*, *Batf* or *NR4A1* gene locus. **d**, **e** ChIP-qPCR assay for TCF1 and SMAD3 at the *Eomes* or *Batf* **d**, *Nr4a1* **e** gene locus in Jurkat cells pre-incubated with Co.EVs, TβRII^+^ (RII+) or TβRII^−^ (RII−) (40 μg) for 48 h. **f** qRT-PCR analysis of indicated T cell exhaustion-related genes in Jurkat cells pre-incubated with TβRII^+^ (RII+) or TβRII^−^ (RII−) (40 μg) for 48 h. **g** Immunoblot analysis of purified CD8^+^ T cells pre-treated with TβRII^+^ (RII+) or TβRII^−^ (RII−) (40 μg). **h** Schematic that depicts the SMAD3 enhances TCF1-dependent transcription. **i** T cell-mediated cancer cell killing assay. MDA-MB-231 cells pre-incubated with Co.EVs or TβRII^+^ (40 μg) were co-cultured for 48 h with or without activated human peripheral blood mononuclear cells (PBMC) in the presence of anti-PD1or anti-PD-L1 antibody at 100 ng/ml. Representative images of crystal violet staining (left) and quantification of cytotoxicity of T cells in different E:T (effector: target) ratio (right). **j** Experimental analysis in vivo: BALB/c mice were nipple injected with 4T1 cells, followed by tail vein-injection of EVs (TβRII^+^ or TβRII^−^ EVs, 50 μg per mouse every other day) and/or anti-PD-1 antibody (100 μg per mouse every other day). Treatment protocol is summarized (left). Tumor volume measured in time (right). Data represent mean ± SD. n = 5 mice per group. **k** Proposed model of the breast cancer EV-TβRII-mediated exhaustion of CD8^+^ T cells and evasion of anti-tumor immunity. Briefly, aggressive breast cancer tumors secrete TβRII^+^ EVs to stimulate TGF-β/SMAD activation in adjacent and remote recipient cells. Up-take of EV-TβRII in the adjacent low-grade tumor cells initiates EMT and increases tumor stemness, drug resistance and metastasis. Meanwhile, EV-TβRII as cargo delivered to CD8^+^ T cells induces the activation of SMAD3 which cooperates with TCF1 transcription factors to impose exhaustion of CD8^+^ T cell and the dysregulation of anti-tumor immunity. Therefore, the release of abundant TβRII^+^ EVs from metastatic breast tumor cells counteract with the anti-tumor immunity systemically. *ns*, not significant (*p* > 0.05) and *p < 0.05 (two-tailed Student’s t test **d**–**f**, **i** or two-way ANOVA **j**). Data are analyzed of three independent experiments and shown as mean + SD **i** or as means ± SD **d**–**f**, **j**. Source data are provided as a Source Data file.

We next investigated the molecular mechanism by which TβRII^+^ TEVs promotes exhaustion of CD8^+^ T cells. Recent studies revealed a key role of TCF1 (T cell factor-1, encoded by *Tcf7*) in establishing exhaustion where the genetic deletion of TCF1 resulted in the loss of exhausted CD8^+^ T cells, specifically during the post-effector phase^48,55^. TβRII^+^ EVs induced sets of transcription factor (TF) changes in CD8^+^ T cells which are correlated with *Tcf7*^56^ (Fig. 8a). Endogenous TCF1 was found to interact with SMAD3 in CD8^+^ T cells pre-treated with TβRII^+^ MDA-MB-231 EVs, but not in cells pre-treated with TβRII^−^ EVs (Fig. 8b, left panel). In vitro purified TCF1 protein associated directly with SMAD3 (Fig. 8b, middle panel), suggesting the activated SMAD3 might modulate TCF1 function. In TGF-β-treated CD8^+^ T cells, endogenous TCF1 also formed complex with SMAD3 (Fig. 8b, right panel).

To investigate potential roles of this binding, we analyzed ChIP-seq data from the Encyclopedia of DNA Elements (ENCODE) project for *Tcf7-correlated* TFs and identified binding motifs for TCF1, SMAD3 and SMAD4. As revealed in genomic browser tracks, binding regions for all three TFs peaks in the T cell exhaustion-associated genes such as *EOMES*, *BATF*, *NR4A1*, *LAG3*, *HIF1α,* and *ID3*, showing that TCF1 co-occupies a higher fraction of loci with SMAD3 (Fig. 8c and Supplementary Fig. 8a). Notably, the binding locations of TCF1 and SMAD3 at *BATF*, *NR4A1* or *ID3* gene locus was associated with a regulatory region highly enriched for H3K27ac signals, indicative of active transcription (Fig. 8c and Supplementary Fig. 8a). We then examined the bindings of TCF1 and SMAD3 to the proximity of these genes upon treatment of either TβRII^+^ or TβRII^−^ EVs. ChIP-qPCR results clearly showed that TCF1 partners with SMAD3 to bind the proximity of those T cell exhaustion-associated genes (Fig. 8d, e and Supplementary Fig. 8b). In line with this, pre-treatment with TβRII^+^ MDA-MB-231 EVs elevated the mRNA expressions of the same set of target genes including *Eomes*, *Batf*, *Nr4a1*, *Tox*, *T-bet*, *Pdcd1* and *Lag3* (Fig. 8f). Moreover, ChIP-qPCR results revealed that TCF1 knockout significantly eliminated the binding of SMAD3 to *Eomes*, *Batf*, *Nr4a1* and *Lag3* genes in the treatment of TβRII^+^ EVs (Supplementary Fig. 8c), indicating that activated SMAD3 indeed regulates those T cell exhaustion-associated genes via binding to TCF1. Western blotting further confirmed that TβRII^+^ TEVs could efficiently induce EOMES expression and reduce GZMB levels in CD8^+^ T cells (Fig. 8g). Collectively, above findings suggested a nodal role of the TCF1 and SMAD3 partnership in mediating CD8^+^ T cell exhaustion (Fig. 8h).

We next evaluated the functional consequences of T cell exhaustion by TβRII^+^ TEVs. For this purpose, T cells from healthy donors were co-cultured with tumor cells at different ratios and were subsequently examined for their tumor-killing efficiency. Strikingly, after incubation with TβRII^+^ TEVs, T cells substantially lost ability to eliminate tumor cell numbers and the lost ability could not be rescued by PD-1 or PD-L1 antibodies (Fig. 8i). In comparison, TβRII^−^ TEVs barely affect the tumor killing capacity of T cells (Fig. 8i). To investigate whether the exogenously introduced EV-TβRII could also antagonize immunotherapy in vivo, we transplanted 4T1 cells in mice followed by tail vein injections of the in vitro collected TEVs from either WT cells or the TβRII null counterparts, combined with or without anti-PD-1 antibody (Fig. 8j). Consistent with our observations in vitro, EV-TβRII suppressed the efficacy of anti-PD-1 antibody in a 4T1-luciferase syngeneic BALB/c model. Tumor size was still increased in both TβRII^+^ TEVs- and anti-PD-1 antibody-treated mice, while the tumor size of mice treated with anti-PD-1 antibody alone reduced significantly (Fig. 8j). Together, above results suggested that breast cancer EV-TβRII promotes tumor growth, metastasis, T cell exhaustion, and immune evasion (Fig. 8k).

Briefly, the aggressive breast cancer tumors secrete TβRII-enriched EVs and stimulate TGF-β/SMAD activation in adjacent pre-malignant tumor cells and remote recipient such as CD8^+^ T cells. Up-take of EV-TβRII in low-grade tumor cells initiate EMT and increase tumor stemness, drug resistance and metastasis. Meanwhile, EV-TβRII as cargo delivered to CD8^+^ T cells induces the activation of SMAD3 which cooperates with TCF1 transcription factors to impose CD8^+^ T cell exhaustion and dysregulation of anti-tumor immunity. Therefore, the contentiously released and rather abundant TβRII^+^ EVs from metastatic breast tumor cells counter the anti-tumor immunity systemically.

## Discussion

Metastatic breast cancers remain largely incurable, even though recent breakthroughs in understanding tumor-immune interplay developed a generation of cancer immunotherapy approaches. Immune checkpoint protein inhibitors, such as antibodies against PD-1 or PD-L1 have shown to have great potential in a limited number of cancer types^57^. However, in patients with malignant breast cancer, the responses are rare^58^, suggesting existence of additional mechanisms used by breast tumors to inhibit host immunity. Our studies suggest that metastatic breast tumor cells release abundant TβRII-positive EVs into the tumor microenvironment and circulation to counter the anti-tumor immunity systemically. Different from TβRI, TβRII is a constitutively active Ser/Thr kinase receptor^59^. Ectopic expression of TβRII, but not TβRI, can strongly stimulate TGF-β/Smad signaling even in the absence of TGF-β ligand and serum, indicating that TβRII as cargo accumulated in recipient cells can stimulate downstream SMAD signaling to occur. The level of TβRII in TEVs is up-regulated by TGF-β, which is frequently present at high levels in aggressive tumors, and the transfer of EV-TβRII could induce SMAD activation in adjacent and remote recipient cells. This unique manner of TGF-β/SMAD regulation provides a concept in TGF-β signaling, particularly in the pathological conditions associated with metastatic breast cancer. In contrast, other TGF-β pathway components, such as SMADs, were not found in the TEVs of breast cancer cells.

In normal and premalignant breast cancer cells where TGF-β inhibits cell proliferation, they express low level of TβRII and EV-TβRII, whereas in high-metastatic breast cancer cells where TGF-β loses tumor suppressive responses and becomes a tumor-promoting factor, they express high level of TβRII and EV-TβRII. TβRII^+^ EVs positively correlated with tumor burden and antagonize immunotherapy. Our results indicate the possible utility of TβRII EVs as markers of metastatic burden and response to therapy. TβRII^+^ EVs could also be used as a selection criterion of anti-TGFβ therapy. We have also established methods for precisely detecting the levels of EV-TβRII and these techniques show that TβRII can be specific marker of extracellular vesicles derived from metastatic breast cancer. Moreover, our studies in orthotopic and spontaneous mouse models suggest that EVs containing TβRII play as a positive regulator of cancer progression. In addition, we have detected efficient up-take of EV-TβRII in low-grade tumor cells, which subsequently initiated EMT and contributed to tumor stemness, drug resistance and increased metastasis. This suggested that TEVs from aggressive tumor cells are capable of transforming their low-grade cells through the TGF-β/SMAD pathway. TβRII is therefore an attractive candidate for detection in EVs present in the circulation of breast cancer patients, in addition to genetic and proteomic analysis of the specific alterations in tumors. Such an approach might enable early detection of breast cancer and help in designing potential curative surgical options.

Breast tumors and their immunoenvironment are interwoven entities^60^. Mice lacking TβRII specifically in T cells display enhanced Th1 responses and strengthened anti-tumor phenotypes, such as production of GZMB and IFN-γ^61–65^. Transgenic mice engineered to express dominant-negative TβRII in CD4^+^ and CD8^+^ T cells are resistant to tumor formation upon inoculation of syngeneic cancer cell lines, which is associated with a large expansion of tumor-reactive CD8^+^ T cells^66^, suggesting that endogenous TβRII could inhibit tumor-antigen-specific T cell priming. The cancer stem cell phenotype is associated with resistance to immunotherapy^67^, so the effect of TβRII^+^ EVs on the tumor cell compartment may also feed into the ineffectiveness of the anti-tumor immune response. In our mice experiments, loss of TβRII in breast tumors was found to associate with reduced exhaustion and increased function of CD8^+^ T cells. In vitro as well as in vivo results showed that TEVs with TβRII as cargo were delivered rapidly and efficiently to CD8^+^ T cells, resulting in activation of their intrinsic TGF-β/SMAD pathway. Mechanistic exploration identified SMAD3 to cooperate with TCF1 transcription factor in regulating T cell exhaustion-associated genes. Binding of TCF1 and SMAD3 indeed coincide with long stretches of H3K27Ac near several genes such as *BATF* and *NR4A1*. Extended H3K27Ac domains have recently been identified and termed super-enhancers and were used to describe regulatory regions that enrich for binding sites of master TFs in the respective cell types^68,69^. Therefore, SMAD3 partners with TCF1 transcription factor to impose CD8^+^ T cell exhaustion and subsequent dysregulation of anti-tumor immunity. Since EV-TβRII-induced T cell exhuastion might be the cause of immunotherapy resistance using either PD-L1 or PD-1 antibodies, our results raise the possibility that reducing EV-TβRII is a previously unrecognized mechanism to improve PD-L1/PD-1 blockade-based therapies.

In conclusion, we have described how breast cancer cells can regulate T cell exhaustion by employing TEVs. By elucidating a TEVs-mediated activation mechanism of TGF-β/SMAD signaling in recipient cells, we have revealed a TEVs-dependent cancer progression mechanism, via which tumor cells can not only become more malignant but also become more repressive to the antitumor immunity, and for which TβRII seems to be the first breast cancer extracellular vesicles marker identified. These mechanistic studies might explain the increased T cell exhuastion frequently found in patients with metastatic breast cancer and offer a therapeutic tool for increasing antitumor immune responses.

## Methods

### Cell culture

HEK293T, HeLa, DR26, HaCaT, MCF7, MDA-MB-231, 4T07 and 4T1 cells were originally from ATCC and cultured in Dulbecco’s modified Eagle’s medium (DMEM) supplemented with 10% FBS (Cat No. FSP500, ExCell Bio) and 100 U ml^−1^ penicillin–streptomycin. B16 cells were from Cell Bank of Type Culture Collection of Chinese Academy of Sciences (Shanghai, China). MCF10A (MI); MCF10A-RAS (MII) cell lines were obtained from Dr. Fred Miller (Barbara Ann Karmanos Cancer Institute, Detroit, USA) and cultured in DMEM/F12 supplemented with 5% horse serum, 20 ng ml^−^^1^ epidermal growth factor (EGF), 0.5 µg ml^−^^1^ hydrocortisone, 100 ng ml^−^^1^ cholera toxin, and 100 U ml^−^^1^ penicillin–streptomycin. All cell lines were cultured in a humidifying atmosphere at 5% CO_2_ at 37 °C and are free of mycoplasma contamination.

### Extracellular vesicles isolation, purification and characterization

For extracellular vesicles purification from cell culture supernatants, cells were plated at a density of 3 million per 10 cm plate and cultured in ‘extracellular vesicles-depleted medium’ (complete medium depleted of FBS-derived extracellular vesicles by overnight centrifugation at 100,000 g) for 48-72 h. The culture media from 10 plates were pooled and subjected to sequential centrifugation steps: 300 × *g* for 5 min at room temperature to remove floating, 2000 × *g* for 15 min at 4 °C to remove dead cells, 16,000 g for 45 min at 4 °C to separate microvesicles, the supernatants were then centrifuged twice at 100,000 × *g* at 4 °C for 2 h as described previously^26^. In each extracellular vesicles/vesicles preparation, the concentration of total proteins was quantified by BCA Protein Assay Kit (Thermo Scientific).

For purification of circulating extracellular vesicles by differential centrifugation, venous citrated blood from breast cancer patients or healthy donors was centrifuged at 1500 g for 20 min at room temperature to obtain cell-free plasma. Then, 1 ml of the obtained plasma was centrifuged at 16,000 g for 45 min at 4 °C to separate microvesicles. The collected supernatants were then centrifuged at 100,000 g for 2 h at 4 °C to pellet the extracellular vesicles. In each extracellular vesicles/vesicles preparation, the concentration of total proteins was quantified by BCA Protein Assay Kit (Thermo Scientific).

The size and concentration of extracellular vesicles/vesicles purified from cell culture supernatants or patients’ plasma were determined using a NanoSight NS500 (Malvern Instruments, Amesbury, UK), which is equipped with fast video capture and particle-tracking software. A monochromatic laser beam at 405 nm was applied to the dilute suspension of vesicles (0.22 μM filtered) with a concentration within the recommended measurement range (1 × 10^6^ to 10 × 10^8^ particles/ml). The Nanoparticle tracking analysis (NTA) software is optimized to first identify and then track each particle on a frame-by-frame basis; the Brownian movement of each particle is tracked and measured from frame to frame. The velocity of particle movement is used to calculate particle size by application of the two-dimensional Stokes-Einstein equation. Nanoparticle tracking analysis (NTA) post-acquisition settings were optimized and kept constant between samples, and each video was then analyzed to give the mean, mode, and median vesicle size together with an estimate of the concentration.

For sucrose density gradient centrifugation (related to Fig. 1e), a discontinuous sucrose gradient (60%, 40%, 20%, 10%, and 5%) was loaded on the bottom, followed by the loading of extracellular vesicles on the top. After the differential centrifugation at 100,000 g for 18 h at 4 °C (Beckman Coulter, Optima MAX-XP). Ten fractions of equal volume were collected from the top of the gradients, with the extracellular vesicles distributed at the density range between 1.13 and 1.19 g/ml. The extracellular vesicles were further pelleted by ultracentrifugation at 100,000 g for 2 h at 4 °C.

### Immunogold labeling and electron microscopy

For verification of purified extracellular vesicles using electron microscopy, purified extracellular vesicles suspended in PBS were dropped on formvar carbon-coated nickel grids. After staining with 2% uranyl acetate, grids were air-dried and visualized using a JEM-1011 transmission electron microscope. For immunogold labeling, purified extracellular vesicles suspended in PBS were placed on formvar carbon-coated nickel grids, blocked, and incubated with TβRII antibody (L-21, Santa Cruz, 1:200) at 4 °C overnight, followed by incubation with the secondary antibody conjugated with anti-rabbit gold antibody (colloidal gold 10 nm, Sigma, G7402, 1:100) for 1 h at RT. Each staining step was followed by five PBS washes and ten ddH_2_O washes before contrast staining with 2% uranyl acetate.

### Patients and specimen collection

Blood samples from healthy donors and patients with breast cancer were collected under the approved sample collection protocol by The First Affiliated Hospital of Zhejiang University and Zhejiang Provincial People’s Hospital Research Ethics Committee. Written consent was obtained from each healthy donor and patient before blood collection. All experiments involving blood samples from healthy donors and patients were performed in accordance with relevant ethical regulations. The tumor grade is known for the tumors at the time of initial plasma collection. The metastasis status in Fig. 3e is the status at the end of follow-up. About the plasma preparation and storage: all post-heparin blood samples were centrifuged at 1500 × *g* for 20 min at RT to collect the platelet-depleted plasma. Plasma stored is frozen at −80 °C before exosome/EV isolation. The plasma was prepared at the time of initial diagnosis/surgery. The plasma was platelet-poor plasma. The collection conditions were consistent according to the standardized experimental procedures and were consistent across this long timeframe.

### Animal studies and mice metastasis models

Mice experiments were approved by the Committee for Animal Welfare at Soochow University. Mice were purchased from the animal husbandry center of the Shanghai Institute of Cell Biology, Academia Sinica, Shanghai, China. Mice were maintained under specific-pathogen-free conditions in the animal facility of Soochow University. The animal room has a controlled temperature (18–23 °C), humidity (40–60%), and a 12 light/12 dark cycle. Experimental/control animals were bred separately. For the intracardial injection assays, five-weeks-old female BALB/c nude were anesthetized with isofluorane and single-cell suspension of MDA-MB-231/luc cells (1 × 10^5^/100 μL PBS) were inoculated into the left heart ventricle according to the method described by Arguello et al.^70^. Bioluminescent imaging was used to verify successful injection and to monitor the metastatic outgrowth. After 6 weeks, all mice were euthanized by cervical dislocation and metastatic lesions were confirmed by histological analysis. For the tail vein injection assay, single-cell suspensions of 4T07-Luc cells (1 × 10^5^/100 μL PBS) cells were injected into the tail vein of five-weeks-old nude mice. Development of metastasis was monitored weekly by bioluminescent reporter imaging. After the indicated days/weeks, all mice were euthanized by cervical dislocation and lung metastases were analyzed. Mouse nipple implantation was based on a previously published method^71^. Female BALB/c mice were anesthetized and used for this assay. A total of 1 × 10^5^ 4T1-Luc cells were injected through the nipple area into the mammary fat pad. 21 days after injection, luciferin was injected and the primary tumors were analyzed, then the mice were euthanized by cervical dislocation and analyzed for acquisition of secondary tumor(s). For B16-OVA experiments, mice were challenged with 1 × 10^5^ B16-OVA tumor cells subcutaneously. Tumors were measured every 2–3 days once palpable using a caliper. Tumor volume was determined by the volume formula for an ellipsoid: 1/2 × *D* × *d*^2^ where *D* is the longer diameter and *d* is the shorter diameter. The mice were euthanized when the tumor size is bigger than 20 mm of the diameter or appeared ulcerated, or mice showed sick or moribund status. Mice were euthanized by cervical dislocation when tumors ulcerated. To deplete CD8^+^ T cells, mice were injected intraperitoneally with 100 µg of anti-CD8α (clone 53–6.7, #BE0004-1, Bio X Cell) or isotype on days -2, -1 (relative to tumor injection on day 0). All primary/metastatic tumors were detected by bioluminescence imaging (BLI) with the IVIS 100 (Caliper Life Sciences, Hopkinton, MA, USA). The BLI signal intensity was quantified as the sum of photons within a region of interest given as the total flux (photons/second). No randomization and blinding were used for mice experiments. For the EVs education experiments, mice were treated with 100 μg EVs in 100 μl PBS via tail vein injection every other day for 3 weeks post tumor injection. For treatment with antibody, 100 mg anti-PD-1 antibody (clone J43, # BP0033-2, Bio X Cell) or rat IgG (Bio X Cell) as control was injected intraperitoneally each mouse every other day after 4T1 cell inoculation.

### Isolation of lymphocytes from mice

To obtain tumor-infiltrating lymphocytes (TIL), excised tumors were harvested, cut into 1 mm^3^ with scissors and digested with 5 ml tumor digestion buffer (5% FBS, 1 mg/ml collagenase IV and 100 µg/mL DNase I). Tissues were digested after rotation for 1 h at 37 °C using the gentle MACS Dissociator (Miltenui Biotec Inc., San Diego, CA, USA). The cell suspension was filtered using a 70-μm filter to obtain a single-cell suspension. TILs were enriched on a Ficoll gradient (Sigma) and washed with DMEM supplemented with 10% FBS. CD8^+^ T cells were isolated by using magnetic beads (Miltenyl Biotec). Lymphocytes from the spleen or lymph nodes were depleted of erythrocytes by hypotonic lysis. Pre-experiments were conducted to ensure that this treatment did not affect expression levels of any of the tested markers.

### Flow cytometry analysis

For analysis of breast cancer stem cell population, cells were prepared according to standard protocols and suspended in 2% inactivated fetal bovine serum with PBS. Antibodies used were against CD24-PE (#562405, BD Bioscience, clone ML5, 1:20) and CD44-APC (#559942, BD Bioscience, clone G44-26, 1:20). For the intracellular cytokine staining, lymphocytes or enriched CD8^+^ T cells were stimulated with plate-bound 1 µg/ml anti-CD3/CD28 and maintained in the presence of 100 U ml^−1^ IL-2 for 24 h prior to intracellular cytokine staining. Then single-cell suspensions were stimulated with 50 ng ml^-1^ Phorbol 12-myristate 13-acetate (PMA) (Sigma, P8139), 1 μM ionomycin (Sigma, I0634), and monensin (ebioscience, 00-4505-51) for 4 h. After stimulation, cells were first stained with anti-CD3e PE (#553063, clone 145-2C11, 1:100, BD), anti-CD4 FITC (#11-0042-82, clone RM4-5, 1:100, eBioscience), anti-CD8 APC (#100712, clone 53-6.7, 1:100, Biolgend), anti-Ly-6G FITC (#11-5931-82, clone RB6-8C5, 1:100, eBioscience), anti-CD11b PE (#12-0112-82, clone M1/70, 1:100, eBioscience), anti-F4/80 FITC (#11-4801-82, clone BM8, 1:100, eBioscience), anti-PD1 FITC (#11-9981-82, clone RMP1-30, 1:100, eBioscience), anti-LAG-3 FITC (#11-2231-82, clone C9B7W, 1:50, eBioscience), anti-TIM3 FITC (#11-5870-82, clone RMT3-23, 1:100, eBioscience) fixed and permeabilized with a Cytofix/Cytoperm kit (554722, BD Biosciences PharMingen) or Foxp3/Transcription Factor Staining Buffer Set (00-5523-00, eBioscience), and finally stained with anti-IFN-γ PE (#12-7311-81, clone XMG1.2, 1:100, eBioscience), anti-TNFα FITC (#11-7321-82, clone MP6-XT22, 1:100, eBioscience), anti-FOXP3 PE (#12-5773-82, clone FJK-16s, 1:50, eBioscience), anti-GZMB FITC (#372206, clone QA16A02, 1:50, Biolgend) in accordance with the manufacturer’s instructions. For the analysis of EVs, 20 μg EVs were mixed with 5 μl 4 μm aldehyde/sulphate latex beads (Invitrogen, no.1743119) in 500 μl 1× PBS for 30 min at room temperature with continuous rotation. EVs-bound beads were incubated with 1 μg anti-TβRII APC-conjugated antibody (#FAB2411A, R&D) for 30 min. The percentage of positive beads was calculated relative to the total number of beads analyzed per sample (10, 000 events). This percentage was therein referred to as the percentage of beads with TβRII^+^ EVs. All samples were analyzed with Beckman CytoFlex (Beckman) or FACSAria II (Becton Dickinson) machines. FACS data were analyzed with CytExpert software and FlowJo v7.6 (TreeStar).

### T cell-mediated tumor cell killing assay

Human peripheral blood mononuclear cells (PBMC) were isolated from healthy donors by Ficoll gradient centrifugation. Briefly, blood was mixed with PBS (1:1) and loaded on lymphocyte separation medium (Cat No. 40504ES60; Yeasen, Shanghai, China) to isolate PBMCs through centrifugation (1,400 g, 25 min). T cells were activated by incubation with anti-CD3 (100 ng/ml, ebioscience, 16-0031-86), anti-CD28 (100 ng/ml, ebioscience, 16-0281-85) and recombinant IL-2 (10 ng/ml, Peprotech, 200-02) in RPMI-1640 (Gibco) for 5–7 d. To analyze the killing of tumor cells by T cells, we co-cultured tumor cells with activated T cells respectively at different ratios according to the purpose of each experiment. After 48–96 h, T cells and cell debris were removed by PBS wash, and living cancer cells were then quantified by a spectrometer at OD (570 nm) followed by crystal violet staining. Anti-human PD1 antibody (clone J116, #BE0188, BioXcell) or anti-human PD-L1 antibody (clone 29E.2A3, #BE0285, BioXcell) were added at a final concentration of 100 ng/ml.

### Lentiviral transduction and the generation of stable cell lines

Lentiviruses were produced by transfecting HEK293T cells with shRNA-targeting plasmids and the helper plasmids pCMV-VSVG, pMDLg-RRE (gag/pol), and pRSV-REV. The cell supernatants were harvested 48 h after transfection and were either used to infect cells or stored at −80 °C.

To obtain stable cell lines, cells were infected at low confluence (20%) for 24 h with lentiviral supernatants diluted 1:1 in normal culture medium in the presence of 5 ng/ml of polybrene (Sigma). At 48 h after infection, the cells were placed under puromycin selection for one week and then passaged before use. Puromycin was used at 2 µg ml^−1^ to maintain MDA-MB-231, MCF10A-RAS, 4T1, and 4T07 derivatives. Lentiviral shRNAs were obtained from Sigma (MISSION® shRNA). Typically, 5 shRNAs were identified and tested, and the two most effective shRNAs were used for the experiment. The sequences of the shRNAs used are listed in Supplementary Table 4. We used the following shRNAs:

TRCN0000197056 (1#) and TRCN0000195606 (#2) for human TβRII knockdown; TRCN0000294600 (1#) and TRCN0000294529 (#2) for mouse TβRII knockdown; TRCN0000279985 (1#) and TRCN0000279982 (#2) for human Rab27a knockdown; TRCN0000040035 (1#) and TRCN0000040036 (#2) for human SMAD2 knockdown; TRCN0000330055 (1#) and TRCN0000330127 (#2) for human SMAD3 knockdown; TRCN0000040030 (1#) and TRCN0000040031 (#2) for human SMAD4 knockdown.

### Transcription reporter assay

HEK293T cells were seeded in 24-well plates and transfected with the indicated plasmids using calcium phosphate. Luciferase activity was measured with a PerkinElmer luminometer. The internal transfection control *renilla*, was used to normalize luciferase activity. Each experiment was performed in triplicate, and the data represent the mean ± SD of three independent experiments.

### Immunoprecipitation and immunoblot analysis

Cells were lysed with 1 ml of lysis buffer (20 mM Tris-HCl pH 7.4, 2 mM EDTA, 25 mM NaF, and 1% Triton X-100) containing protease inhibitors (Sigma) for 10 min at 4 °C. After centrifugation at 12 × 10^3^ × *g* for 15 min, the protein concentrations were measured, and equal amounts of lysate were used for immunoprecipitation. Immunoprecipitation was performed with anti-FLAG M2 beads (Sigma, A2220) for 1 h at 4 °C or with different antibodies and protein A/G-Sepharose (GE Healthcare Bio-Sciences AB) for 3 h at 4 °C. Thereafter, the precipitants were washed three times with washing buffer (50 mM Tris-HCl pH 8.0, 150 mM NaCl, 1% Nonidet P-40, 0.5% sodium deoxycholate, and 0.1% SDS), and the immune complexes were eluted with sample buffer containing 1% SDS for 5 min at 95 °C. The immunoprecipitated proteins were then separated by SDS-PAGE. Immunoblot (IB) analysis was performed with specific antibodies and secondary anti-mouse or anti-rabbit antibodies conjugated to horseradish peroxidase (Amersham Biosciences). Visualization was achieved with chemiluminescence. For proteins that migrated close to the IgG heavy chain, protein A-HRP (horseradish peroxidase) was used. For the analysis of cell surface receptors, the proteins at the cell surface were biotinylated for 40 min at 4 °C and then incubated at 37 °C for the indicated times. The biotinylated cell surface receptors were precipitated with streptavidin beads and analyzed by immunoblotting.

The antibodies used for immunoprecipitation (IP), immunoblotting (IB), immunofluorescence (IF), and immunohistochemistry (IHC) were as follows: TβRII (sc-400, Santa Cruz Biotechnology, 1:1000 for IB, 1:50 for IP, 1:100 for IHC, 1:50 for immunogold label, 1:100 for IF), human TβRII (ab184948, Abcam, 1:1000 for IB), CD63 (ab216130, Abcam, 1:2000 for IB), TSG101 (sc-7964, Santa Cruz Biotechnology, 1:1000 for IB), Alix (sc-53540, Santa Cruz Biotechnology, 1:1000 for IB, 1:100 for IF), CD9 (A19027, ABclonal, 1:2000 for IB), CD81(sc-166029, Santa Cruz Biotechnology, 1:1000 for IB), Calnexin (A4846, ABclonal, 1:2000 for IB), Hrs (A1790, ABclonal, 1:2000 for IB), Rab27a (sc-74586, Santa Cruz Biotechnology, 1:1000 for IB), N-cadherin (610920, BD Bioscience, 1:50000 for IB), E-cadherin (610181, BD Bioscience, 1:10000 for IB, 1:100 for IF), SMAD4 (sc-7966, Santa Cruz Biotechnology, 1:1000 for IB), SMAD2-3 (610842, BD Bioscience, 1:2500 for IB, 1:500 for IP, 1:100 for IF), phospho-SMAD2 (#3101, Cell Signaling, 1:5000 for IB, 1:50 for IHC), SMAD3 (A19115, ABclonal, 1:100 for IP, 1:2000 for IB), phospho-SMAD3 (AP0727, ABclonal, 1:2000 for IB), SMAD4 (A19116, ABclonal, 1:2000 for IB), Ub (sc-8017, Santa Cruz Biotechnology, 1:1000 for IB), fibronectin (SAB4500974, Sigma, 1:1000 for IB), SMA (SAB5500002, Sigma, 1:1000 for IB), vimentin (#5741, Cell Signaling, 1:1000 for IB), TCF1/7 (#2203, Cell Signaling, 1:1000 for IB), EOMES (#4540, Cell Signaling, 1:1000 for IB), GZMB (sc-8022, Santa Cruz Biotechnology, 1:1000 for IB), CD8a (ab22378, Abcam, 1:400 for IHC), K48-linkage specific polyubiquitin (#8081 S, Cell Signaling, 1:1000 for IB), K63-linkage specific polyubiquitin (#A18164, ABclonal, 1:1000 for IB), β-actin (#A5441, Sigma, 1:10000 for IB), Phalloidin (#93042, Sigma, 1:1000 for IF), polyclonal HA(Y-11) (sc-805, Santa Cruz Biotechnology, 1:1000 for IB), monoclonal HA(12CAS5, home-made, 1:5000 for IB), polyclonal Myc (A-14) (sc-789, Santa Cruz Biotechnology, 1:1000 for IB), monoclonal Myc (9E10) (sc-40, Santa Cruz Biotechnology, 1:1000 for IB), Flag (M2, Sigma, 1:10000 for IB, 1:200 for IF), Protein A–HRP (Sigma-Aldrich GENA9120, 1:10000 for IB), HRP-conjugated secondary antibodies to mouse (NA931) or rabbit (NA934) (both from Amersham Biosciences, 1:10000 for IB), AlexaFluor488-labeled secondary antibody to rabbit (Molecular Probes R37116, 1:300 for IF) or AlexaFluor593-labeled antibody to mouse (Molecular Probes R3712, 1:300 for IF).

### Immunofluorescence

Cells grown on glass coverslips were washed with PBS and fixed with 4% paraformaldehyde in PBS for 20 min, permeabilized with 0.2% Triton X-100 and blocked with 3% bovine serum albumin. Then the cells were stained with indicated antibodies (identified above), followed by incubation with fluorescent-dye-conjugated secondary antibodies (identified above). Nuclei were counterstained with DAPI (Sigma-Aldrich).

### CRISPR-Cas9-mediated genome editing

CRISPR/Cas9 genomic editing for gene deletion or replacement was used, as previously described^72^. For deletion of the gene encoding TβRII, CRISPR guide RNA (sgRNA)s were cloned into the vector lentiCRISPRv2 (addgene) and transfected into target cells. 48 h after transfection, cells were placed under puromycin selection for one week and the single clones were picked, grown and identified by immunoblot and sequencing. The guide RNA sequences that were used are as follows:

mouse *TβRII* sg1, 5′-AACGTGCGGCGGGATCGTGC-3′;

mouse *TβRII* sg2, 5′-TGCTGGCGATGCGCGTCCAC-3′;

human *TβRII* sg, 5′-AACGTGCGGTGGGATCGTGC-3′.

### Gene set enrichment analysis (GSEA)

We used GSEA v2.0 to perform GSEA on various functional and/or characteristic gene signatures. Gene sets were obtained from the MSigDB database v3.0 (September 2010 release). Statistical significance was assessed by comparing the enrichment score to enrichment results generated from 1000 random permutations of the gene set to obtain *P* values (nominal *P* value).

### Primers and reagents

DNA primer sequences used to detect target gene expression by qRT-PCR are listed in Supplementary Table 4. TβRII was amplified by standard PCR with GolddenSatr® T6 Super PCR Mix (TSE101, Tsingke Biotechnology Co., Ltd) and cloned in pEGFP-N1 and pLV-Flag vector. Point mutations were generated by the site-directed mutagenesis with KOD plus (Toyobo) polymerase. All constructs were confirmed by DNA sequencing. shRNAs were obtained from the Sigma Mission Library: TRCN0000294600 for mouse TβRII was subcloned into Tet-pLKO-puro (#21915, Addgene) for DOX inducible knockdown. CHX was from Sigma (C104450); SB431542 was from Millipore (616461); TGF-β (10804) and TβRII-ICD (10358) were from Sino Biological Inc.; GW4869 was from MCE (HY-19363); MG132 was from SelleckChem. CFSE Cell Division Tracker Kit was purchased from Biolgend (# 423801).

### Real-time RT-PCR (qRT-PCR)

Total RNAs were prepared using the NucleoSpin® RNA II kit (BIOKÉ, Netherlands). A total of 1 μg of RNA was reverse-transcribed using the HiScript® II Q RT SuperMix ((R223-01, Vazyme Biotech co., ltd). Real-time PCR was conducted with ChamQ Universal SYBR qPCR Master Mix (Q711-02, Vazyme Biotech co., ltd). All target gene expression levels were normalized to *GAPDH*. The primers used for qRT-PCR are listed in Supplementary Table 5.

### Chromatin immunoprecipitation-quantitative PCR (ChIP-qPCR)

ChIP assays were performed according to the standard cross-linking ChIP protocol (Abcam) with minor modifications. Briefly, approximately 10 million cells were harvested and crosslinked with 1% formaldehyde for 10 min at room temperature. After sonication, the soluble chromatins were incubated with specific antibodies. Chromatin immunocomplex was then precipitated with Protein A. The immunoprecipitated complex was washed, and DNA was extracted and purified by QIAquick PCR Purification Kit (Qiagen). Antibodies used for ChIP-qPCR were as follows: TCF1 (Cell Signaling, #2203 S, 1:50), SMAD3 (Cell Signaling, #9523 S, 1:50), and IgG (Santa Cruz). After the purification of DNA, Quantitative real-time PCR (qRT-PCR) was conducted with SYBR Green (Applied Biosystems) using a StepOne Plus real-time PCR system (Applied Biosystems) and specific primers against the genomic regions of interest. The data were normalized by input DNA and the results were derived from three independent experiments. The primers used for qRT-PCR and ChIP-qPCR are listed in Supplementary Table 5. Publically available CTCF ChIP-seq datasets of the ENCODE consortium^73^ were downloaded from UCSC (http://genome.ucsc.edu/ENCODE) for Homo sapiens HepG2 cells (ENCSR444LIN, ENCSR005GZH, ENCSR826YMT, and ENCSR000AMO).

### Protein purification and in vitro binding assay

For in vitro protein-protein interactions, purified and/or recombinant proteins were diluted and mixed in binding buffer (25 mM HEPES [pH 7.5], 100 mM KCl, 2 mM MgCl_2_, 0.1% NP40, 5% glycerol), immunoprecipitated with anti-antibody for 3 h and protein A-Sepharose beads for 1 h, and washed three times with the same buffer. The precipated material was then analyzed for coprecipitating proteins by immunoblotting.

### Tumor sphere assays

Single cell suspensions of MCF10A (RAS) cells (1 × 10^3^ cells ml^−1^) were plated on ultra-low attachment plates and cultured in phenol red-free DMEM/F12 (Gibco Paisley, UK; 21041) containing B27 supplement (no vitamin A; Invitrogen, Paisley, UK; 12587) and rEGF (20 ng/ml; Sigma Aldrich Poole, UK; E-9644). Tumor spheres were visualized using a phase contrast microscope, photographed, and counted.

### 3D spheroid invasion assays

Semi-confluent MCF10A-RAS cells were trypsinized, counted, and re-suspended in medium containing 2.4 mg/ml methylcellulose (Sigma) at the concentration of 10^4^ cells ml^−1^. A total of 100 μl of suspension was added into each well of U-bottom 96-well-plate allowing the formation of one spheroid per well. All spheroids consisted of 10^3^ cells. Two days after plating, spheroids were harvested and embedded into collagen. Flat-bottom 96-well-plate was coated with neutralized bovine collagen-I (PureCol, Advanced BioMatrix) according to manufacturer’s protocol. Single spheroids were embedded in a 1:1 mix of neutralized collagen and medium supplemented with 12 mg/ml of methylcellulose. Invasion was monitored during the next two days and quantified by measuring the area occupied by cells using ImageJ software. Pictures were taken at day 0, day 1, day 1.5 after embedding.

### Affinity capture of biotinylated proteins

Cells were washed with 1 × PBS and lysed in 2 ml lysis buffer (50 mM Tris pH 7.4, 500 mM NaCl, 0.1% SDS, 5 mM EDTA, 1 mM DTT) containing protease inhibitors. Then the lysates were briefly sonicated. After centrifugation at 16 × 10^3^ g for 10 min, Biotin-Ub-VME probes were added in the supernatants at 4 °C for 3 h to target the active DUBs, the cell lysates were then incubated with 50 μl neutravidin beads at 4 °C overnight. After extensive washing, bound proteins were eluted with 2 × SDS sample buffer and separated on SDS-PAGE followed by western blotting.

### Mass spectrometry

SDS-PAGE gels were minimally stained with Coomassie brilliant blue, cut into 6 molecular weight ranges based on heavy chain IgG bands, and digested with trypsin. Immunocomplexes were identified on a Thermo Fisher LTQ (majority) or Velos-Orbitrap mass spectrometer. Spectral data were then searched against the human protein RefSeq database in BioWorks or the Proteome Discoverer Suites using either SeQuest (for LTQ data) or Mascot (Orbitrap data) software. The IP/MS results were transferred into a FileMaker-based relational database generated in-house, where protein identification numbers (protein GIs) were converted to GeneID identifiers according to the NCBI “gene accession”.

### Enzyme-linked immunosorbent assay (ELISA)

The concentrations of EV-TβRII in culture supernatants and plasma were measured by ELISA Kits (EHTGFBR2, Invitrogen). The concentrations of total active TGF-β1 in plasma of breast cancer patients were measured by ELISA Kits (MM-0090H1, MEIMIAN). Briefly, blood from breast cancer patients or healthy donors was centrifuged at 1,300 g for 20 min at room temperature to obtain platelet-free plasma. The efficient activation of TGF-β1 was obtained after reduction of the pH to 1.5 by addition of 5 M HCl and neutralization with 1.4 M NaOH in 0.7 M Hepes. All experiments were performed according to the manufacturer’s instructions.

### Cell viability assay

Cell viability was analyzed by 3-(4,5-dimethylthiazol-2-yl)-2,5 diphenyltetrazolium bromide (MTT) assay. Briefly, 5 × 10^3^ cells were seeded into each well of 24-well culture plates for overnight followed by treatment of individual component for 3 days. MTT was added to cells for 4 h, MTT formazan crystals were solubilized with DMSO and read at 560 nm on a micro plate reader.

### Immunohistochemical staining and evaluation

Primary antibodies to p-SMAD2 (1:50; Cell Signaling, #3108) was used for immunohistochemical stainings, according to previously described staining protocols^74^. Briefly, tissue specimens were incubated with antibodies and a biotin-conjugated secondary antibody and then mixed with an avidin-biotin-peroxidase complex. Amino-ethylcarbazole chromogen was used for visualization. The quantification of protein expression was obtained by measuring the *H* score. The H-score was calculated as: the formula 3 × the percentage of strongly staining cells + 2 × the percentage of moderately staining cells + the percentage of weakly staining cells, yielding an H-score range of 0 to 300.

### Statistical analyses

Data are shown as mean ± s.d. and statistical analyses were performed with a two-tailed unpaired Student’s t-test or as indicated in the figure legends. Statistical differences with a *P* value of 0.05 or less were considered significant. The exact value of *n*, representing the number of mice in the experiments, is indicated in the figure legends. For mouse survival studies, Kaplan–Meier survival curves were generated and analyzed for statistical significance with GraphPad Prism 7.0 & 8.0. Pilot studies were used for the estimation of the sample size to ensure adequate power. There was no exclusion of data points or mice. No randomization or blinding was used.

### Reporting summary

Further information on research design is available in the Nature Research Reporting Summary linked to this article.

## Supplementary information

Supplementary Information

Reporting Summary

## Data Availability

Publically available CTCF ChIP-seq datasets of the ENCODE consortium were downloaded from UCSC (http://genome.ucsc.edu/ENCODE) for Homo sapiens HepG2 cells. ENCSR444LIN, hg38, can be accessed using the link. ENCSR005GZH, hg38, can be accessed using the link. ENCSR826YMT, hg38, can be accessed using the link. ENCSR000AMO, hg38, can be accessed using the link. Source data for Figs. 1–8, and Supplementary Figs. 1–8 are provided with this paper as a Source Data file. The remaining data are available within the Article, Supplementary Information or Source Data file. Source data are provided with this paper.

## Source data

Source Data
