# Supplementary material for: Breast cancer cell-derived extracellular vesicles promote CD8+ T cell exhaustion via TGF-β type II receptor signaling
Source: Nat Commun. 2022 Aug 1;13:4461. doi: 10.1038/s41467-022-31250-2 (PMC9343611; doi:10.1038/s41467-022-31250-2)
Supplement: Supplementary file 1 — Supplementary Information [file 41467_2022_31250_MOESM1_ESM.pdf]

Supplementary Information

Breast Cancer Cell-derived Extracellular Vesicles Promote CD8+ T cell Exhaustion via TGF-β type II receptor Signalling

Feng Xie, Xiaoxue Zhou, Peng Su, Heyu Li, Yifei Tu, Jinjin Du, Chen Pan, Xiang Wei, Min Zheng, Ke Jin, Liyan Miao, Chao Wang, Xuli Meng, Hans van Dam, Peter ten Dijke, Fangfang Zhou [¶], Long Zhang [¶]

[¶] Corresponding authors: F.Z. (zhoufangfang@suda.edu.cn) or L.Z. (L_Zhang@zju.edu.cn)

This PDF file includes:

Supplementary Figures 1-8 and Supplementary Table 1-5.

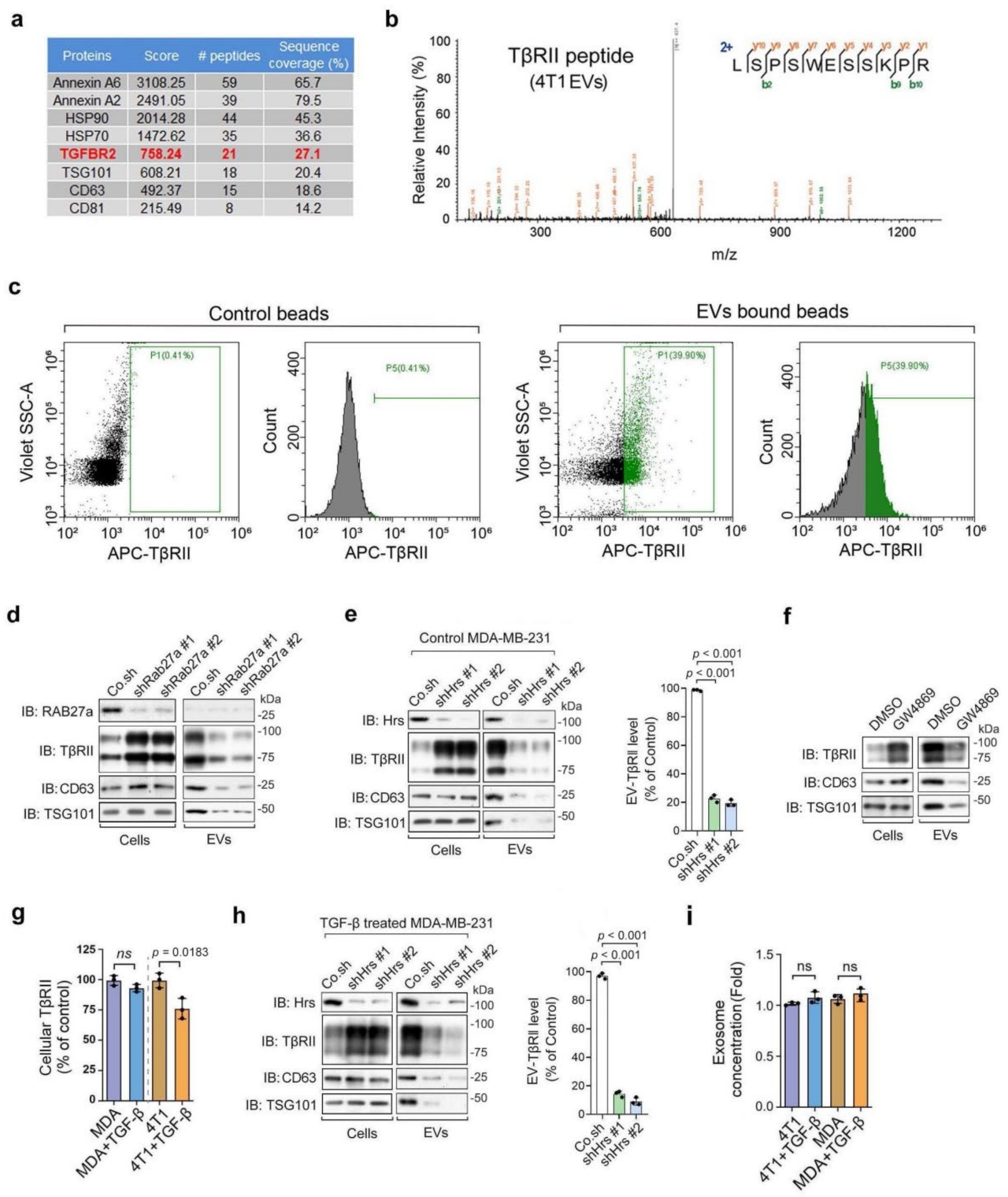

Supplementary Figure 1. Related to Figure 1; Malignant breast cancer cells release EVs carrying TβRII.

a, Mass-spectrometry analysis of purified EVs secreted from 4T1 cells, showing results for Annexin A2, Annexin A6, HSP90, HSP70, TGFBR2 (red), TSG101, CD63 and CD81. **b**, TβRII peptide identified by mass-spectrometry analysis of purified EVs from 4T1 cells. **c**,The gating strategy for the FACS beads-bound EVs assay. **d**, Immunoblot analysis of TβRII in whole cells lysate and EVs in MDA-MB-231 cells upon RAB27A knockdown. **e**, Immunoblot analysis (left) and quantification (right) of

TβRII in whole cells lysate and EVs of MDA-MB-231 cells infected with lentivirus encoding control (Co.sh) or Hrs shRNA (# 1 and # 2). **f**, Immunoblot analysis of TβRII in whole cells lysate and EVs from MDA-MB-231 cells with GW4869 (10 μM) treatment for 48 h. **g**, Quantification of TβRII in whole cells lysate from control cells and TGF-β-treated cells in Fig. 1h. **h**, Immunoblot analysis (left) and quantification (right) of TβRII in whole cells lysate and EVs of MDA-MB-231 cells infected with lentivirus encoding control (Co.sh) or Hrs shRNA (# 1 and # 2) with TGF-β treatment for 16 h. **i**, EVs concentration by nanosight of 4T1 and MDA-MB-231 cells without or with TGF-β treatment (5 ng/ml) for 48 h.

ns, not significant ($p > 0.05$) and $^*p < 0.05$ (unpaired two-tailed Student's t test (**e**, **g**, **h**, **i**)). Data are analyzed of three independent experiments and shown as mean ± SD (**e**, **g**, **h**, **i**). Source data are provided as a Source Data file.

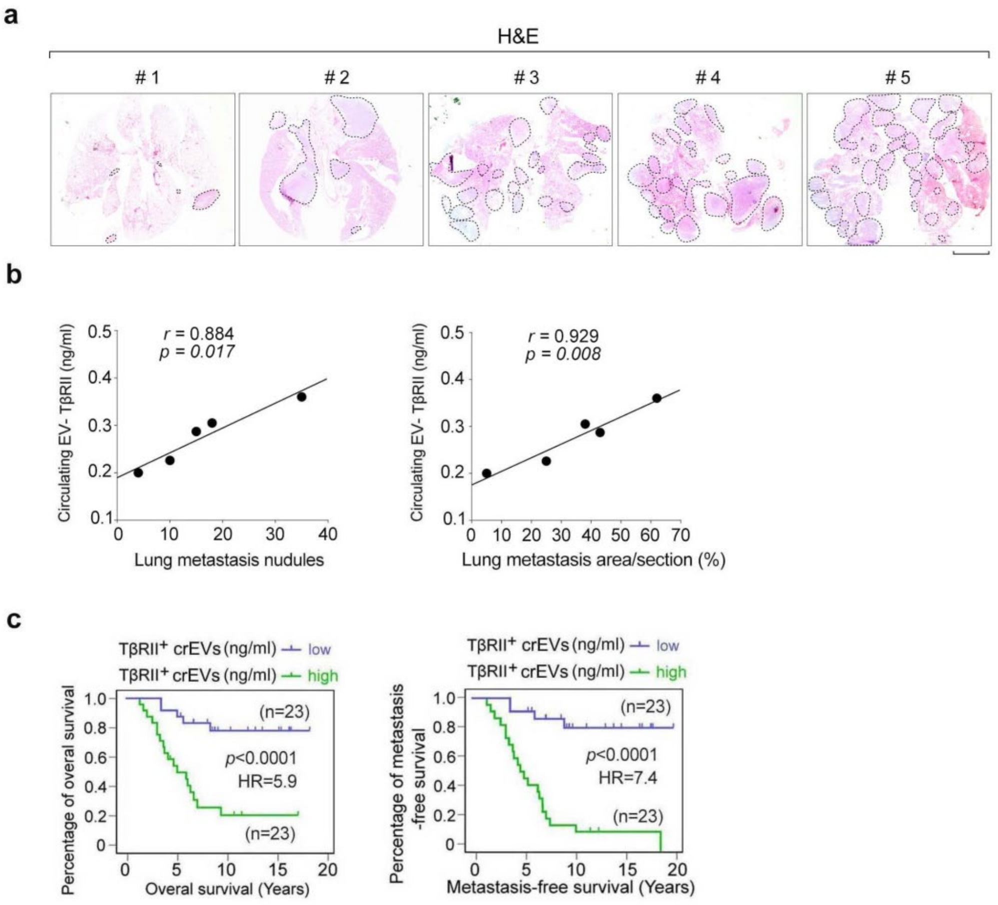

Supplementary Figure 2. Related to Figure 2 and Figure 3; EV-TβRII is a biomarker for breast cancer.

a-b, HE stained lung sections of all mice (**a**), scale bars, 2 μm; pearson correlation between the level circulating EV-TβRII in plasma and the numbers of lung metastasis nodules (left) or lung area occupied by metastases (right) in MMTV-PyMT mice (n = 5) (**b**) in Fig. 2e. **c**, Kaplan–Meier curves (log-rank test) displaying overall (left) and metastasis free (right) survival of patients with a high (>1.1 ng/ml, ELISA-detected level) $T\beta RII^+$ crEVs (green), and with a low (<1.1 ng/ml, ELISA-detected level) $T\beta RII^+$ crEVs (blue).

*$p < 0.05$ (two-way ANOVA (**b**)). Source data are provided as a Source Data file.

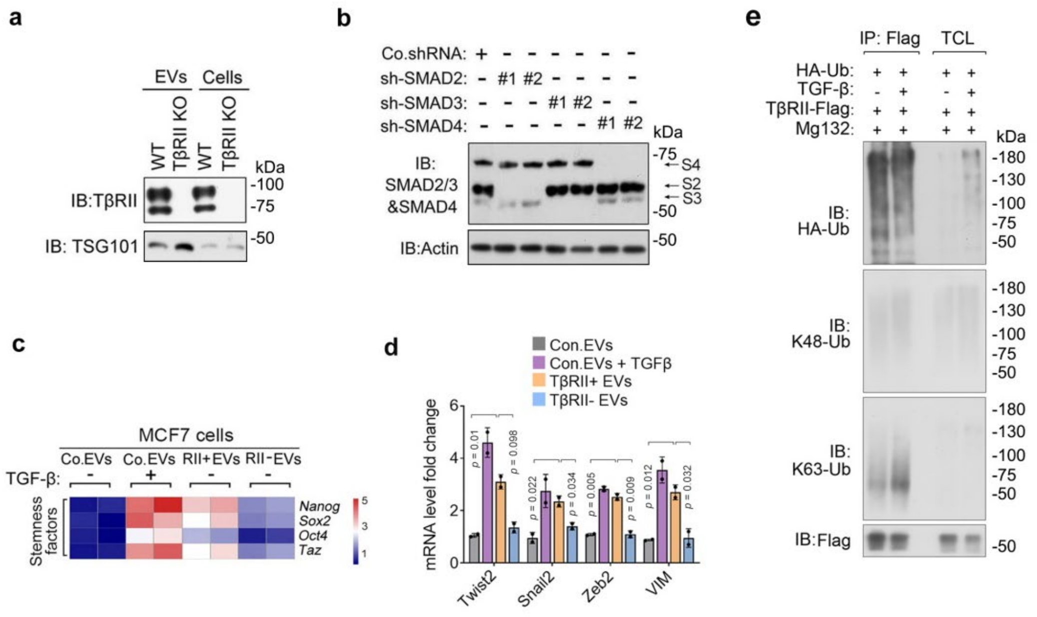

Supplementary Figure 3. Related to Figure 4; EV-TβRII mediates TEV-induced SMAD activation and breast cancer stemness.

a, Immunoblot analysis of TβRII in total cells lysate and EVs derived from control or TβRII-knockout MDA-MB-231 cells. **b**, Immunoblot analysis of 293T cells infected with lentivirus encoding control shRNA (Co.sh), SMAD2 shRNA (#1, #2), SMAD3 shRNA (#1, #2) or SMAD4 shRNA (#1, #2). **c**, qPCR analysis in MCF7 cells pre-incubated for 48 h with Co.EVs, $T\beta RII^{+}$ or $T\beta RII^{-}$ EVs (40 µg), followed by no stimulation (-) or stimulation (+) for 16 h with TGF-β (2.5 ng/ml). Relative mRNA levels are shown as a heatmap. **d**, qPCR analysis in MCF7 cells pre-incubated for 48 h with Co.EVs, $T\beta RII^{+}$ or $T\beta RII^{-}$ EVs (40 µg), followed by no stimulation (-) or stimulation (+) for 16 h with TGF-β (2.5 ng/ml). **e**, Immunoblot of immunoprecipitants of EVs derived from HA-Ub stably expressed HEK293T cells transfected with TβRII-Flag and treated without or with TGF-β (5 ng/ml) for 24h and MG132 (5 µM) for 6 h.

*$p < 0.05$ (unpaired two-tailed Student's t test (**d**)). Data are analyzed of two independent experiments and shown as mean + SD (**d**). Source data are provided as a Source Data file.

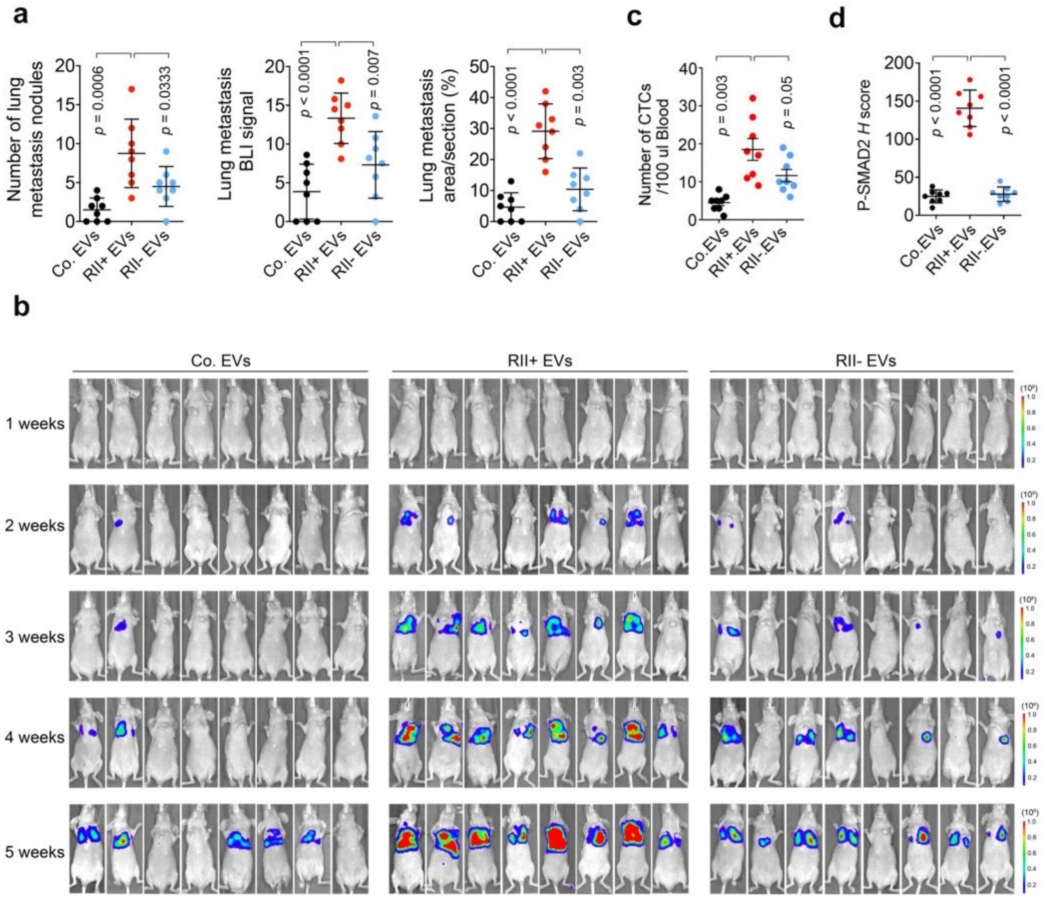

Supplementary Figure 4. Related to Figure 5; TβRII^{+} EVs regulate metastatic outgrowth of breast cancer cells *in vivo.*

a, Number of lung metastasis nodules (left), BLI signals of lung metastasis (middle) and lung metastasis area (right) of all mice in each experimental group (n = 8 mice per group) at week 5 were shown. **b**, The complete set of BL images of all mice in Fig. 5j (n = 8 mice per group). **c**, Circulating tumor cells in each group were measured by bioluminescence assay upon 4-week after injection. **d**, H Score of P-SMAD2 protein from each group was evaluated.

p* < 0.05 (unpaired two-tailed Student's t test (a**, **c**, **d**)). Data are analyzed of three independent experiments and shown as means ± SD (**a**, **c**, **d**). Source data are provided as a Source Data file.

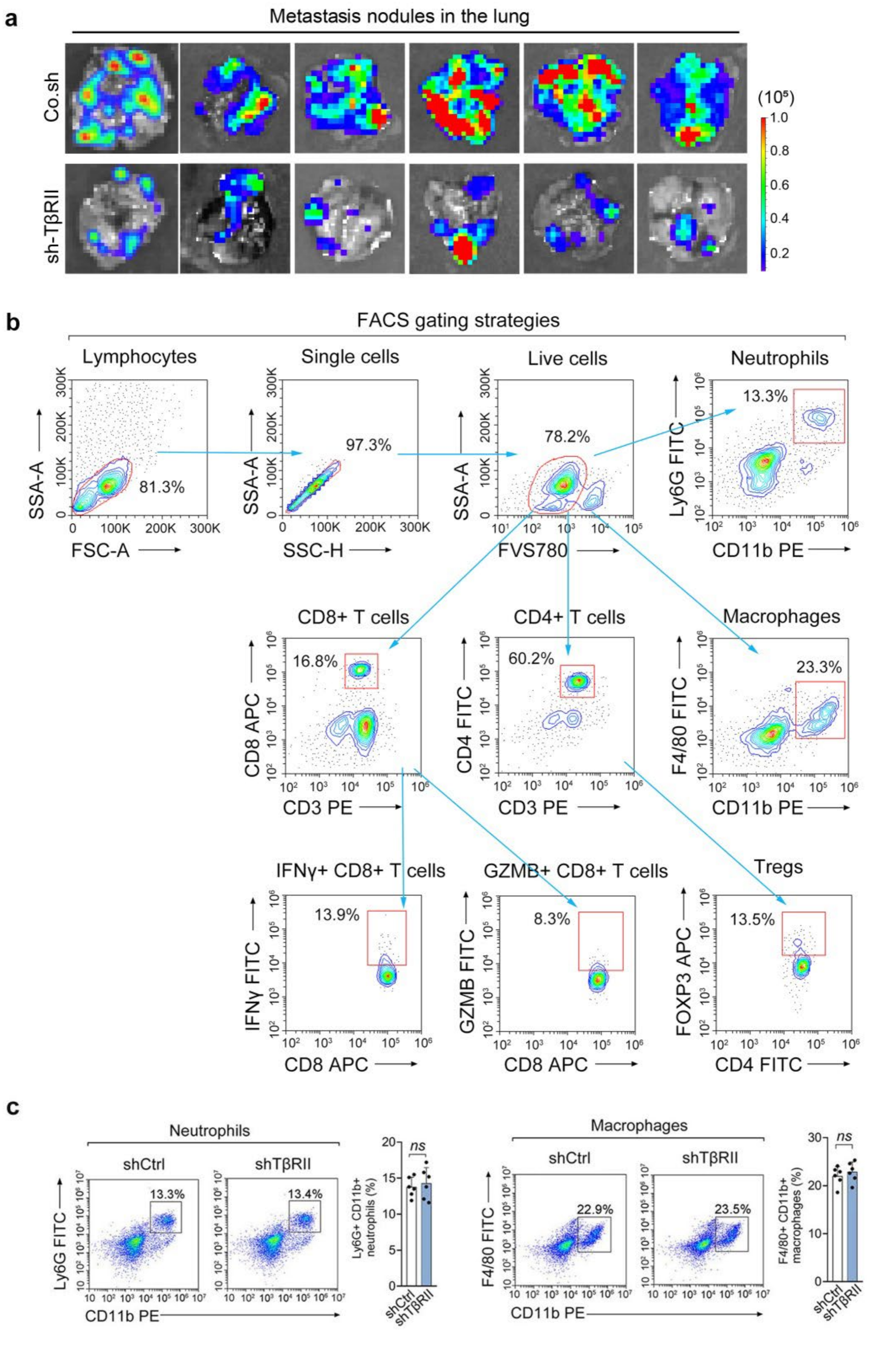

Supplementary Figure 5. Related to Figure 6; TβRII^{+} crEVs promote metastasis and have no effects on neutrophils and macrophages.

a, The complete set of BL images of metastasis nodules in the lung of all mice in Fig.

6e. **b**, Flow Cytometry gating strategies for $CD8^+$ T cells, $CD4^+$ T cells, neutrophils, macrophages, $IFN\gamma^+$ $CD8^+$ T cells, $GZMB^+$ $CD8^+$ T cells and Tregs. **c**, FACS analysis and quantification of the percentage of neutrophils (left, n = 6 mice per group, $p = 0.6335$) and macrophages (right, n = 6 mice per group, $p = 0.4911$) from spleen.
ns, not significant ($p > 0.05$) (unpaired two-tailed Student's t test (**c**)). Data are shown as mean+ SD (**c**). Source data are provided as a Source Data file.

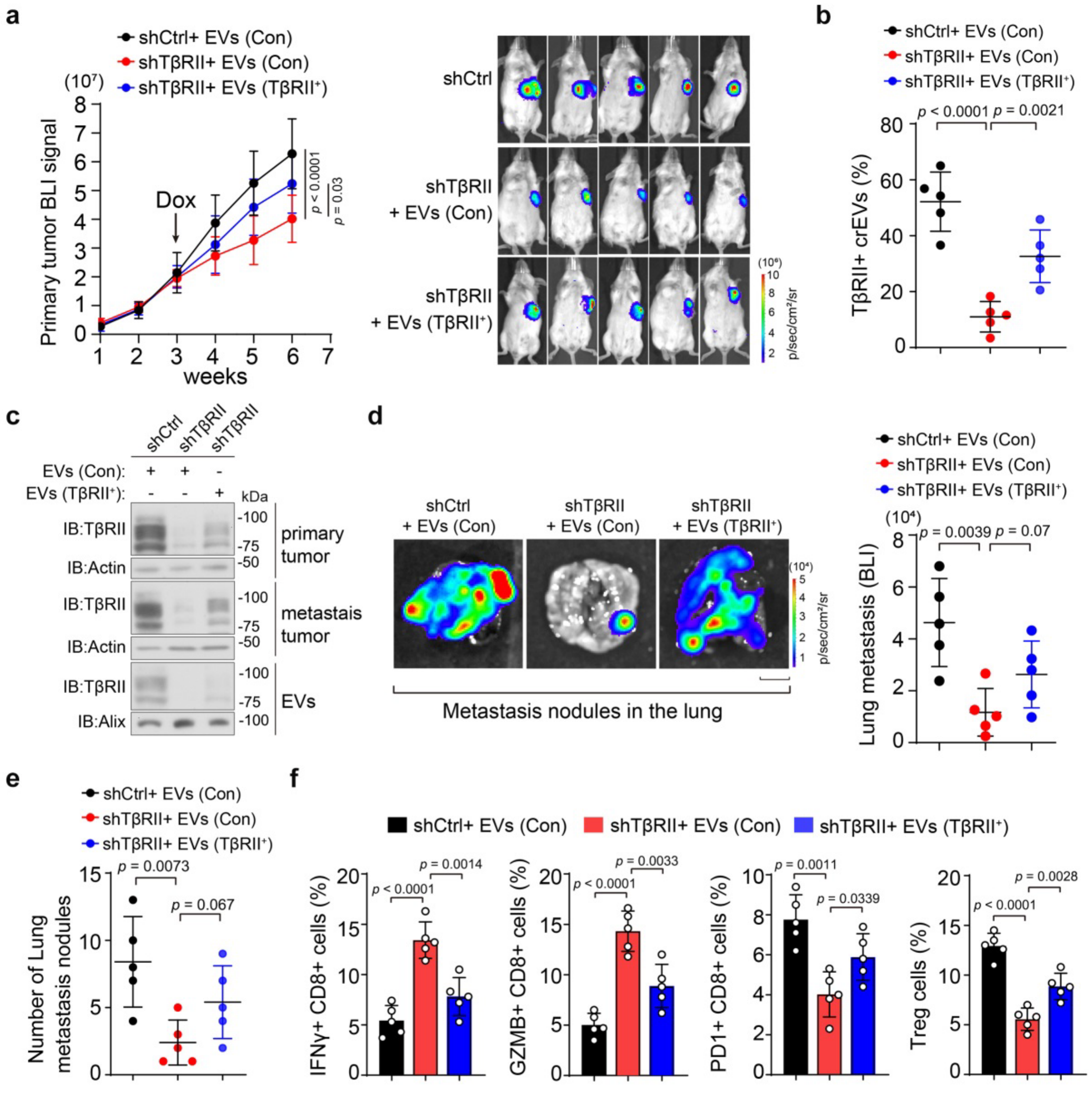

Supplementary Figure 6. Related to Figure 6; TβRII^{+} crEVs rescue the tumor growth rate and metastasis in the absence of TβRII *in vivo*.

a-f, Experimental analysis *in vivo*: BALB/c mice were nipple injected with 4T1 cells (2×10^5 cells per mouse) expressing control shRNA or doxycycline-inducible shRNA targeting TβRII (shTβRII) and tumors were grown for 3 weeks, followed by the administration of doxycycline (Dox) and tail vein-injection of TβRII^{+} or control EVs (100 µg) three times per week for 3 weeks (n = 5 mice per group). BLI signals (left) from each group at the indicated times and BLI imaging of representative mice at week 6 (right) (**a**). Percentage of TβRII+ crEVs in plasma of all mice in each group at week 6 (**b**). Immunoblot analysis of TβRII in primary tumor, metastatic tumor and

circulating EVs from plasma in mice (**c**). Representative bioluminescent view in each group (left) and BLI signals (right) of lung metastasis. Scale bar, 2 mm (**d**). Number of lung metastasis nodules (**e**). Quantification of the percentage of IFNγ$^+$,GZMB+ and PD1$^+$ of CD8$^+$ cells and Treg cells from tumor-infiltrating lymphocyte (TIL) populations from all mice in each group at week 6 (**f**).

*$p < 0.05$ (unpaired two-tailed Student's t test (**b-f**) or two-way ANOVA (**a**)). Data are analyzed of three independent experiments and shown as mean ± SD (**a, b-f**). Source data are provided as a Source Data file.

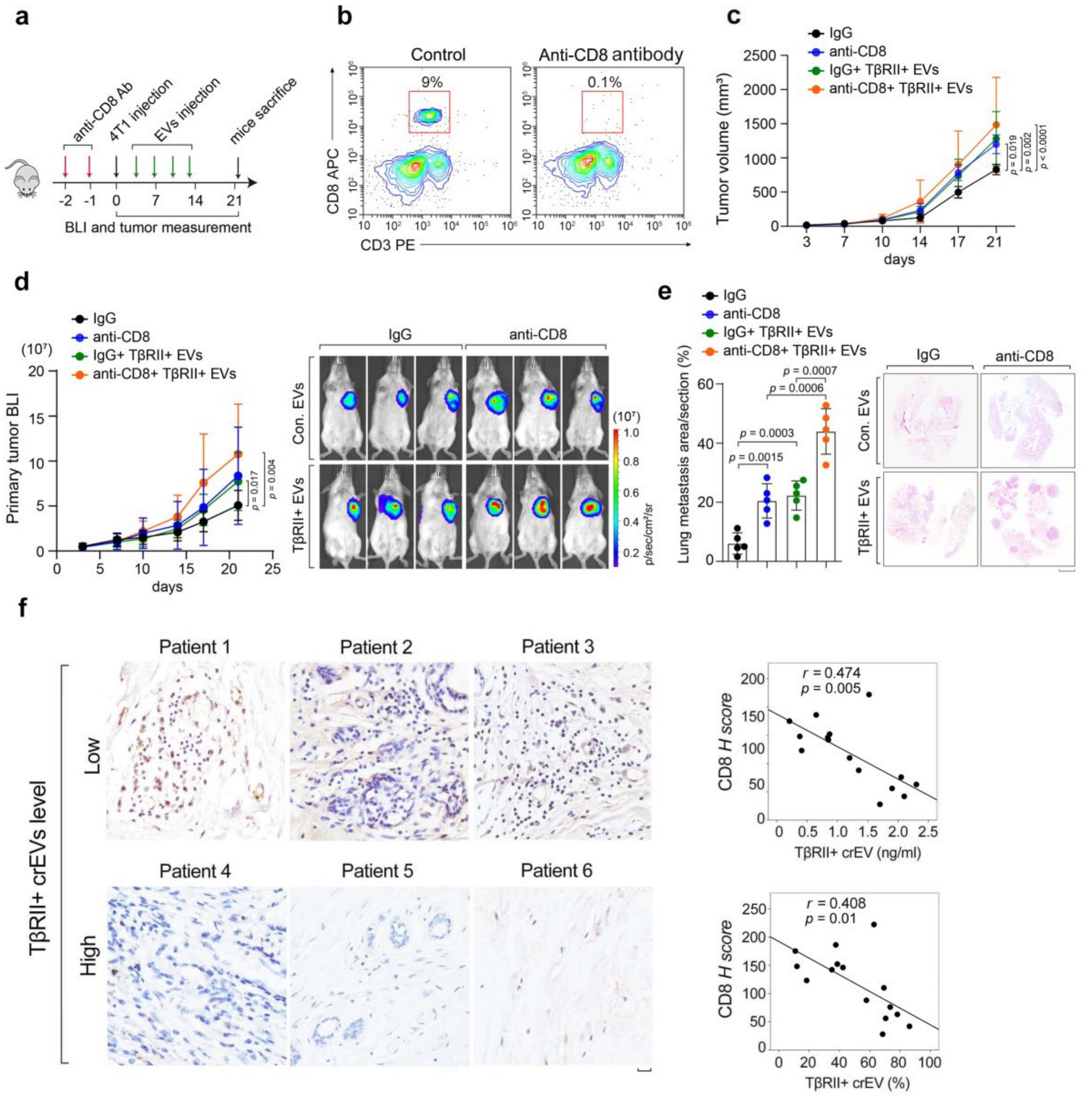

Supplementary Figure 7. Related to Figure 6; EV-TβRII inhibits anti-tumor immunity in mice.

a-e, Experimental analysis *in vivo*: BALB/c mice were intraperitoneally injected with anti-CD8 antibody (100 µg per mouse) for two days before 4T1-luc cells nipple inoculation (2×10^5 cells per mouse), followed by tail vein-injection of TβRII+ or control EVs (100 µg) twice per week for 2 weeks (n = 5 mice per group) (**a**). FACS analysis of the percentage of CD8+ T cells in plasma samples from mice at week 3 (**b**). Tumor volume measurement of mice from each group at the indicated times (**c**). BLI signals of primary tumor from each group at the indicated times (left) and BLI imaging of representative mice at week 3 (right) (**d**). Tumor metastases area per HE

stained lung section and representative HE stained lung sections from each group at week 3. Scale bar, 2 mm (**e**). **f**, Immunohistochemical staining of CD8a in representative breast cancer specimens (n = 15 samples) with low or high level of TβRII+ crEVs in plasma. Brown staining indicataes positive immunoreactivity (left). Scatterplot showing the negative correlation between the TβRII+ crEVs level and the tumor-infiltrating CD8+ T level in patients (right). Scale bars, 20 μm.

ns, not significant ($p > 0.05$) and *$p < 0.05$ (unpaired two-tailed Student's t test (**e**) or two-way ANOVA (**c**, **d**, **f**)). Data are analyzed of three independent experiments and shown as mean ± SD (**c, d, e**). Source data are provided as a Source Data file.

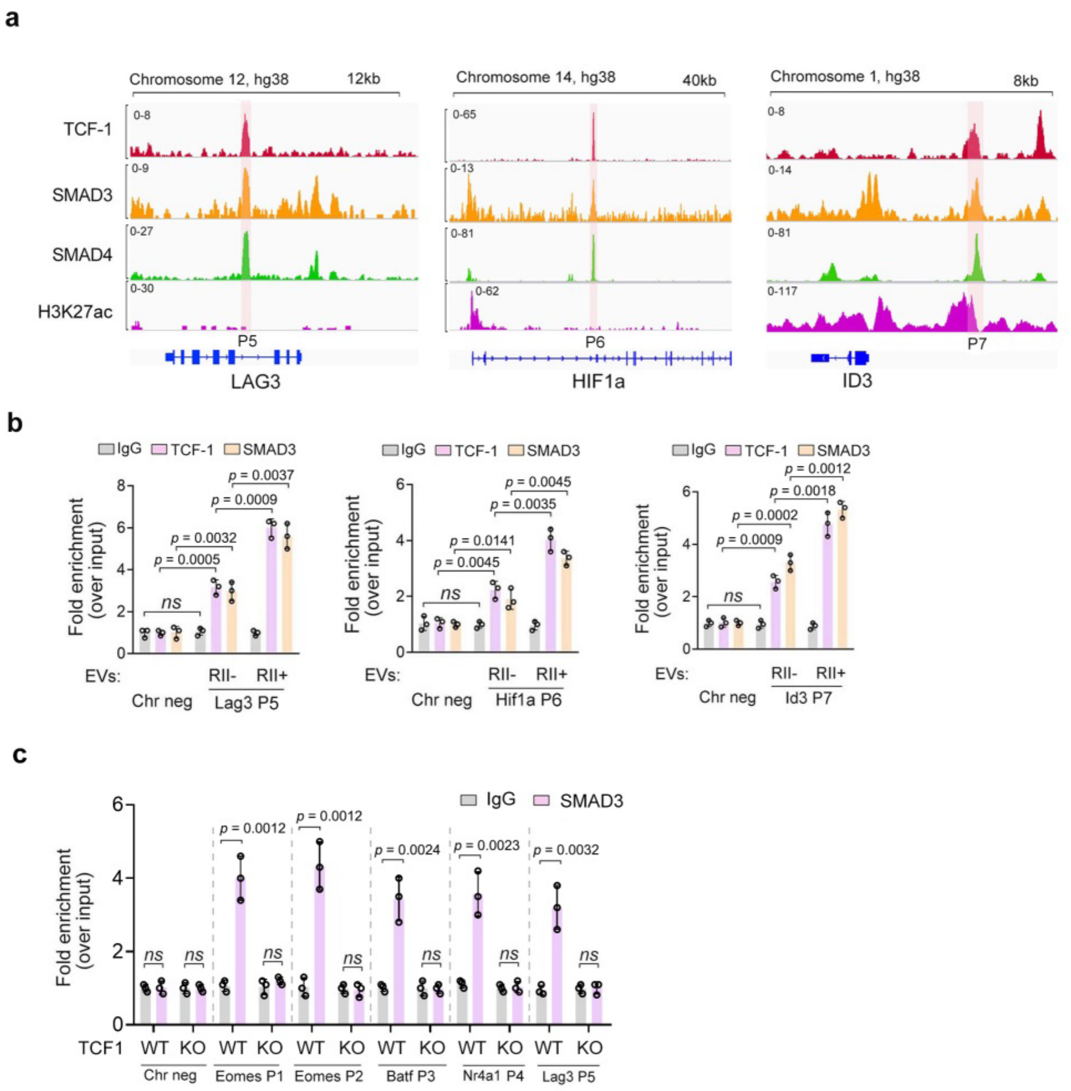

Supplementary Figure 8. Related to Figure 8; TCF1 partners with SMAD3 to regulate T cell exhaustion-associated genes.

a, Genome Browser tracks from ENCODE in HepG2 cells representing the binding sites of TCF1, SMAD3, SMAD4 and H3K27ac at the *Lag3*, *Hif1α* or *Id3* gene locus in HapG2 cells. **b**, ChIP-qPCR assay for TCF1 and SMAD3 at the *Lag3*, *Hif1a* and *Id3* gene locus in Jurkat cells pre-incubated with Co.EVs, TβRII^{+} (RII+) or TβRII^{-} (RII-) (40 μg) for 48 h. **c**, ChIP-qPCR assay for SMAD3 at the *Eomes*, *Batf*, *Nr4a1*and *Lag3* gene locus in control and TCF1 knockout Jurkat cells pre-incubated with TβRII^{+} EVs for 48 h.

ns, not significant ($p > 0.05$) and $*p < 0.05$ (unpaired two-tailed Student's t test (**b**, **c**)).

Data are analyzed of three independent experiments and shown as mean+ SD (**b**, **c**). Source data are provided as a Source Data file.

Supplementary Table S1 Detailed data of Breast Cancer patients

Number	IFN-γ (pg/ml)	TβRII⁺ crExos (ng/ml)	TβRII⁺ crExos (%)	Event Death	Time Survival	Event Meta	Time Recurrence	Time Meta	Subtypes	Tumor grade
1	87.61	0.60	23.21	0	15.52	0	15.52	0	Luminal A	Ⅰ
2	167.88	0.18	4.21	0	16.30	0	16.30	0	Luminal A	Ⅰ
3	119.58	0.12	9.30	0	8.21	0	8.21	0	Luminal B	Ⅱ
4	191.31	0.08	6.21	0	12.21	0	12.21	0	Luminal B	Ⅰ
5	110.32	0.38	11.97	0	13.65	0	13.65	0	Luminal B	Ⅰ
6	72.63	1.35	57.80	1	6.25	1	6.25	0	Luminal B	Ⅲ
7	108.41	0.27	28.24	0	9.25	0	9.25	0	Luminal A	Ⅱ
8	88.75	0.84	42.32	0	6.85	0	6.85	0	Luminal A	Ⅲ
9	66.07	0.70	31.60	0	8.92	0	8.92	0	Luminal B	Ⅱ
10	111.17	0.21	10.89	1	5.22	0	1.12	0	Luminal A	Ⅲ
11	53.23	1.42	61.00	1	3.85	1	3.50	3.5	HER2+	Ⅲ
12	134.27	0.15	15.52	0	16.35	0	16.35	0	Luminal B	Ⅰ
13	86.53	0.41	18.35	0	6.80	0	5.60	0	Luminal A	Ⅱ
14	42.58	1.15	63.52	1	3.95	1	3.50	3.5	HER2+	Ⅲ
15	63.86	0.76	43.85	0	8.70	0	8.70	0	TNBC	Ⅲ
16	74.26	1.45	54.35	1	7.20	1	3.50	7	TNBC	Ⅲ
17	53.86	0.85	35.02	0	8.95	0	8.95	0	HER2+	Ⅲ
18	84.23	0.99	52.41	0	12.96	0	12.96	0	HER2+	Ⅱ
19	67.28	0.83	36.52	0	5.56	0	5.56	0	HER2+	Ⅲ
20	48.21	2.30	78.21	1	2.20	1	1.50	1.5	TNBC	Ⅳ
21	35.12	1.22	56.25	1	6.52	1	6.52	3.6	HER2+	Ⅲ
22	28.12	1.52	62.80	0	6.55	1	6.55	6.55	TNBC	Ⅲ
23	16.23	1.20	69.35	0	4.52	1	2.80	4.3	HER2+	Ⅲ
24	62.12	1.10	51.93	1	9.55	1	3.68	3.68	HER2+	Ⅱ
25	51.23	1.42	56.58	1	2.75	1	1.72	1.72	HER2+	Ⅲ
26	45.25	1.00	49.80	0	18.35	0	18.35	0	TNBC	Ⅰ
27	35.21	0.57	46.32	0	16.50	0	16.50	0	TNBC	Ⅱ
28	28.36	1.55	58.50	1	1.50	1	0.98	0.98	HER2+	Ⅳ
29	31.25	1.70	68.52	0	17.20	1	10.50	10.5	TNBC	Ⅱ
30	17.65	2.05	73.50	1	3.20	1	1.10	1.1	TNBC	Ⅳ
31	24.12	1.07	40.56	0	10.85	0	10.85	0	TNBC	Ⅱ
32	25.36	1.85	79.24	1	6.85	1	6.85	4.25	TNBC	Ⅲ
33	76.35	1.90	70.50	1	5.20	1	4.20	4.2	TNBC	Ⅲ
34	45.86	1.62	82.41	1	4.85	1	2.50	4.5	TNBC	Ⅲ
35	34.16	2.10	86.24	1	3.50	1	2.50	2.5	TNBC	Ⅳ
36	86.42	1.80	83.25	1	1.85	1	0.90	0.9	TNBC	Ⅳ
37	65.31	0.56	25.80	0	10.50	0	10.50	0	HER2+	Ⅱ
38	45.62	1.22	40.30	1	4.30	1	2.70	2.7	TNBC	Ⅲ
39	36.15	1.43	51.80	1	6.10	1	3.50	3.5	TNBC	Ⅲ
40	32.69	1.15	31.50	1	3.60	1	1.60	1.6	HER2+	Ⅲ
41	22.14	1.68	70.80	1	3.20	1	1.80	1.8	TNBC	Ⅳ
42	71.54	0.72	45.60	0	6.90	1	5.80	5.8	TNBC	Ⅲ
43	102.56	0.25	19.50	0	15.30	0	15.30	0	Luminal B	Ⅰ
44	58.87	0.65	37.60	0	13.10	0	13.10	0	HER2+	Ⅱ
45	41.26	1.25	61.50	0	11.60	0	11.60	0	TNBC	Ⅱ
46	90.32	0.87	38.60	1	8.50	1	6.30	6.3	TNBC	Ⅲ

Supplementary Table S2 Detailed data of healthy donors

Number	TβRII^{+} crExos (ng/ml)	TβRII^{+} crExos (%)
1	0.075	2.600
2	0.130	6.300
3	0.223	8.240
4	0.041	7.214
5	0.063	4.582
6	0.175	15.252
7	0.120	7.240
8	0.150	9.250
9	0.038	1.935
10	0.070	3.251
11	0.170	4.520
12	0.105	3.854
13	0.088	4.850
14	0.145	6.350
15	0.063	3.214
16	0.160	12.524
17	0.190	10.250
18	0.030	3.140
19	0.112	5.854
20	0.115	6.850

Supplementary Table S3 Detailed data associated with the ROC curve

Parameter	AUC	Cut-off	Sensitivity	Specificity	95% CI	Accuracy	Positive predictive value	Negative predictive value
TβRII$^+$ crEVs (%)	0.872	>10.707 %	93.48 % (43/46)	90 % (18/20)	0.789-0.956	92.42 % (61/66)	95.55 % (43/45)	85.71 % (18/21)
TβRII$^+$ crEVs(ng/ml)	0.967	>0.236 ng/ml	89.13 % (41/46)	100 % (20/20)	0.930-1.000	92.42 % (61/66)	100 % (41/41)	80 % (20/25)

Supplementary Table S4 the sequences of the shRNAs used

		Target Sequence		Oligo design
human TβRII shRNAs	TRCN0000197056 (#1)	GACCTCAAGAGCTCCAATATC	F	5'-CCGGGACCTCAAGAGCTCCAATATCCTCGAGGATATTGGAGCTCTTGAGGTCTTTTTG-3'
			R	5'-AATTCAAAAAGACCTCAAGAGCTCCAATATCCTCGAGGATATTGGAGCTCTTGAGGTC-3'
	TRCN0000195606 (#2)	CGACATGATAGTCACTGACAA	F	5'-CCGGCGACATGATAGTCACTGACAACTCGAGTTGTCAGTGACTATCATGTCGTTTTTG-3'
			R	5'-AATTCAAAAACGACATGATAGTCACTGACAACTCGAGTTGTCAGTGACTATCATGTCG-3'
mouse TβRII shRNAs	TRCN0000294600 (#1)	GAAGGACATCTTCTCCGATAT	F	5'-CCGGGAAGGACATCTTCTCCGATATCTCGAGATATCGGAGAAGATGTCCTTCTTTTTG-3'
			R	5'-AATTCAAAAAGAAGGACATCTTCTCCGATATCTCGAGATATCGGAGAAGATGTCCTTC-3'
	TRCN0000294529 (#2)	CCAGATCGTGTGTGAGACTTT	F	5'-CCGGCCAGATCGTGTGTGAGACTTTCTCGAGAAAGTCTCACACACGATCTGGTTTTTG-3'
			R	5'-AATTCAAAAACCAGATCGTGTGTGAGACTTTCTCGAGAAAGTCTCACACACGATCTGG-3'
human Rab27a shRNAs	TRCN0000279985 (#1)	CCAGTGTACTTTACCAATATA	F	5'-CCGGCCAGTGTACTTTACCAATATACTCGAGTATATTGGTAAAGTACACTGGTTTTTG-3'
			R	5'-AATTCAAAAACCAGTGTACTTTACCAATATACTCGAGTATATTGGTAAAGTACACTGG-3'
	TRCN0000279982 (#2)	CGGATCAGTTAAGTGAAGAAA	F	5'-CCGGCGGATCAGTTAAGTGAAGAAACTCGAGTTTCTTCACTTAACTGATCCGTTTTTG-3'
			R	5'-AATTCAAAAACGGATCAGTTAAGTGAAGAAACTCGAGTTTCTTCACTTAACTGATCCG-3'
human SMAD2 shRNAs	TRCN0000040035 (#1)	GCGTTGCTCAAGCATGTCATA	F	5'-CCGGGCGTTGCTCAAGCATGTCATACTCGAGTATGACATGCTTGAGCAACGCTTTTTG-3'
			R	5'-AATTCAAAAAGCGTTGCTCAAGCATGTCATACTCGAGTATGACATGCTTGAGCAACGC-3'
	TRCN0000040036 (#2)	CGATTAGATGAGCTTGAGAAA	F	5'-CCGGCGATTAGATGAGCTTGAGAAACTCGAGTTTCTCAAGCTCATCTAATCGTTTTTG-3'
			R	5'-AATTCAAAAACGATTAGATGAGCTTGAGAAACTCGAGTTTCTCAAGCTCATCTAATCG-3'
human SMAD3 shRNAs	TRCN0000330055 (#1)	GCCTCAGTGACAGCGCTATTT	F	5'-CCGGGCCTCAGTGACAGCGCTATTTCTCGAGAAATAGCGCTGTCACTGAGGCTTTTTG-3'
			R	5'-AATTCAAAAAGCCTCAGTGACAGCGCTATTTCTCGAGAAATAGCGCTGTCACTGAGGC-3'
	TRCN0000330127 (#2)	GAGCCTGGTCAAGAAACTCAA	F	5'-CCGGGAGCCTGGTCAAGAAACTCAACTCGAGTTGAGTTTCTTGACCAGGCTCTTTTTG-3'
			R	5'-AATTCAAAAAGAGCCTGGTCAAGAAACTCAACTCGAGTTGAGTTTCTTGACCAGGCTC-3'
human SMAD4 shRNAs	TRCN0000040030 (#1)	GCTGCTGGAATTGGTGTTGAT	F	5'-CCGGGCTGCTGGAATTGGTGTTGATCTCGAGATCAACACCAATTCCAGCAGCTTTTTG-3'
			R	5'-AATTCAAAAAGCTGCTGGAATTGGTGTTGATCTCGAGATCAACACCAATTCCAGCAGC-3'
	TRCN0000040031 (#2)	CGAGTTGTATCACCTGGAATT	F	5'-CCGGCGAGTTGTATCACCTGGAATTCTCGAGAATTCCAGGTGATACAACTCGTTTTTG-3'
			R	5'-AATTCAAAAACGAGTTGTATCACCTGGAATTCTCGAGAATTCCAGGTGATACAACTCG-3'

Supplementary Table S5 Primers list

RT-qPCR		
Gene	Forward	Reverse
EOMES	5'-CCACTGCCCACTACAATGTG-3'	5'-TTCCCGAATGAAATCTCCTG-3'
BATF	5'-GCGAAGACCTGGAGAAACAG-3'	5'-GGAGCTGACATGAGGTTGGT-3'
NR4A1	5'-GGCATGGTGAAGGAAGTTGT-3'	5'-CGGAGAGCAGGTCGTAGAAC-3'
TOX	5'-CTGCCTCTGATATGGGGAAA-3'	5'-CATTGATGCCACAATCTTCG-3'
T-bet	5'-CCGTGACTGCCTACCAGAAT-3'	5'-ATCTCCCCCAAGGAATTGAC-3'
PDCD1	5'-GTGTCACACAACTGCCCAAC-3'	5'-CTGCCCTTCTCTCTGTCACC-3'
LAG3	5'-ATGGCTTCAACGTCTCCATC-3'	5'-CTTGGCAGTGAGGAAAGACC-3'

ChIP-qPCR		
EOMES-1	5'-AACACCGCCTGCACCGGT-3'	5'-TATGATAAGGCATTCCTA-3'
EOMES-2	5'-AAGGGGGCCCATATAAAT-3'	5'-ATTAAAGCTCCCTCCCTC-3'
BATF	5'-ATCAGCCAGGAACAGTTC-3'	5'-AAGTGCTGGGATTACAGG-3'
NR4A1	5'-GGCTAGGCTCGGAGGGAG-3'	5'-AGGCGGCCAGGGGGAGGT-3'
LAG3	5'-GTCTGGGAAGTTAGAAGGAA-3'	5'-ATGTTCACGT ATTCATTCAA-3'
HIF1α	5'-GTGAAATGCCGTCTCAGG-3'	5'-CAGGCTCTTT CTTGGAGT-3'
ID3	5'-CAAATGCCTCTGAACTTT-3'	5'-GCCGGTGACTGCCCGAGG-3'
