# Supplementary material for: Breast cancer cell-derived extracellular vesicles promote CD8+ T cell exhaustion via TGF-β type II receptor signaling
Source: Nat Commun. 2022 Aug 1;13:4461. doi: 10.1038/s41467-022-31250-2 (PMC9343611; doi:10.1038/s41467-022-31250-2)
Supplement: Supplementary file 2 — Reporting Summary [file 41467_2022_31250_MOESM2_ESM.pdf]

nature research

Corresponding author(s): Fangfang Zhou

Last updated by author(s): Apr 12, 2022

# Reporting Summary

Nature Research wishes to improve the reproducibility of the work that we publish. This form provides structure for consistency and transparency in reporting. For further information on Nature Research policies, see our Editorial Policies and the Editorial Policy Checklist.

## Statistics

For all statistical analyses, confirm that the following items are present in the figure legend, table legend, main text, or Methods section.

| n/a | Confirmed | |
|---|---|---|
| ☐ | ☒ | The exact sample size ($n$) for each experimental group/condition, given as a discrete number and unit of measurement |
| ☐ | ☒ | A statement on whether measurements were taken from distinct samples or whether the same sample was measured repeatedly |
| ☐ | ☒ | The statistical test(s) used AND whether they are one- or two-sided<br>*Only common tests should be described solely by name; describe more complex techniques in the Methods section.* |
| ☒ | ☐ | A description of all covariates tested |
| ☒ | ☐ | A description of any assumptions or corrections, such as tests of normality and adjustment for multiple comparisons |
| ☐ | ☒ | A full description of the statistical parameters including central tendency (e.g. means) or other basic estimates (e.g. regression coefficient) AND variation (e.g. standard deviation) or associated estimates of uncertainty (e.g. confidence intervals) |
| ☐ | ☒ | For null hypothesis testing, the test statistic (e.g. $F$, $t$, $r$) with confidence intervals, effect sizes, degrees of freedom and $P$ value noted<br>*Give P values as exact values whenever suitable.* |
| ☒ | ☐ | For Bayesian analysis, information on the choice of priors and Markov chain Monte Carlo settings |
| ☒ | ☐ | For hierarchical and complex designs, identification of the appropriate level for tests and full reporting of outcomes |
| ☐ | ☒ | Estimates of effect sizes (e.g. Cohen's $d$, Pearson's $r$), indicating how they were calculated |

*Our web collection on statistics for biologists contains articles on many of the points above.*

## Software and code

Policy information about availability of computer code

Data collection

1. Flow cytometric data were collected using Beckman CytoFlex (Beckman).
2. Enzyme linked Immunosorbent Assay data were collected using BioTek Synergy Neo2 Multi-Mode Reader.
3. Immunofluorescence images were acquired using a ZEISS LSM 880 (Zeiss) confocal microscope.
4. qPCR were performed using Bio-Rad CFX-96 Touch.
5. Mass Spectrometry was performed on a Thermo Fisher QE-HFX mass spectrometer.
6. Bioluminescent reporter imaging was obtained using IVIS® Spectrum In Vivo Imaging System.
7. The extracellular vesicles were analyzed using a NanoSight NS500 (Malvern Instruments, Amesbury, UK).
8. The transmission electron microscopy (TEM) images were taken with a JEOL JEM-1011 transmission electron microscope.
9. Luciferase activity was quantified using a Centro XS3 LB960 illuminometer (Berthold Technologies, Bad Wildbad, Germany).

Data analysis

1. Statistical analysis was performed with GraphPad Prism (version 7.0 & 8.0) and SPSS (version 19.0).
2. FACS data was analyzed with CytExpert software (version 2.3) and FlowJo software (version 7.6).
3. Spectral data was analyzed by Proteome Discovery software (version 1.4) and MaxQuant (version 1.6).
4. The ChIP-seq datasets were visualized by The Integrative Genomics Viewer (IGV) software (version 2.9.4).
5. Gene Set Enrichment Analysis was performed using the GSEA software (version 2.0).
6. The size and concentration of extracellular vesicles were analyzed by Nanoparticle tracking analysis (NTA) software (version 3.0).
7. Immunofluorescence images were analyzed by Zen software blue version 2.3.
8. Western blot bands were analyzed by Image J software (version 1.8.0)

For manuscripts utilizing custom algorithms or software that are central to the research but not yet described in published literature, software must be made available to editors and reviewers. We strongly encourage code deposition in a community repository (e.g. GitHub). See the Nature Research guidelines for submitting code & software for further information.

## Data

Policy information about availability of data

All manuscripts must include a data availability statement. This statement should provide the following information, where applicable:

- Accession codes, unique identifiers, or web links for publicly available datasets
- A list of figures that have associated raw data
- A description of any restrictions on data availability

Publically available CTCF ChIP-seq datasets of the ENCODE consortium were downloaded from UCSC (http://genome.ucsc.edu/ENCODE) for Homo sapiens HepG2 cells. ENCSR444LIN, hg38, can be accessed using the link https://www.encodeproject.org/experiments/ENCSR444LIN/. ENCSR005GZH, hg38, can be accessed using the link https://www.encodeproject.org/experiments/ENCSR005GZH/. ENCSR826YMT, hg38, can be accessed using the link https://www.encodeproject.org/experiments/ENCSR826YMT/. ENCSR000AMO, hg38, can be accessed using the link https://www.encodeproject.org/experiments/ENCSR000AMO/. The authors declare that all other data that support the findings of this study are available within the paper, its supplementary information, or Source Data file.

# Field-specific reporting

Please select the one below that is the best fit for your research. If you are not sure, read the appropriate sections before making your selection.

☒ Life sciences ☐ Behavioural & social sciences ☐ Ecological, evolutionary & environmental sciences

For a reference copy of the document with all sections, see nature.com/documents/nr-reporting-summary-flat.pdf

# Life sciences study design

All studies must disclose on these points even when the disclosure is negative.

| | |
|---|---|
| Sample size | For in vivo studies, n=5-10 mice per group is sufficient to detect meaningful biological differences with good reproducibility. For signaling assays, we typically performed at least three independent experiment repeats to allow statistical analysis and robust conclusions to be drawn. We determined the sample size to be sufficient based on literature (PMID: 29358709, 28443643, and 30472188) and the lab's previous experience. |
| Data exclusions | No data were excluded. |
| Replication | All the findings were reliably reproduced in multiple independent experiments. For all experiments, our data represent at least two independent assays that produce similar results. We also used different assays and readouts to confirm our findings in different way. |
| Randomization | Animals used were randomly assigned to each treatment group. No particular randomization method was used for other experiments. The same number of cells were plated for each treatment condition and same amount of protein was used for co-IP and immunoblotting analyses for each experimental condition. |
| Blinding | For the in vivo studies, collection of data was blinded, but to calculate differences between treatment groups each animal number had to be matched to its treatment. For the analyses using patient samples, the investigators were blinded to group allocation during data collection, the type of response was matched to each sample number during data analysis. For histological analyses, slides were blinded prior to scoring. All other experiments were performed in a nonblinded manner, because the experimental design was complicated and blinding feasibility was poor. |

# Reporting for specific materials, systems and methods

We require information from authors about some types of materials, experimental systems and methods used in many studies. Here, indicate whether each material, system or method listed is relevant to your study. If you are not sure if a list item applies to your research, read the appropriate section before selecting a response.

## Materials & experimental systems

| n/a | Involved in the study | |
|---|---|---|
| ☐ | ☒ | Antibodies |
| ☐ | ☒ | Eukaryotic cell lines |
| ☒ | ☐ | Palaeontology and archaeology |
| ☐ | ☒ | Animals and other organisms |
| ☐ | ☒ | Human research participants |
| ☒ | ☐ | Clinical data |
| ☒ | ☐ | Dual use research of concern |

## Methods

| n/a | Involved in the study | |
|---|---|---|
| ☒ | ☐ | ChIP-seq |
| ☐ | ☒ | Flow cytometry |
| ☒ | ☐ | MRI-based neuroimaging |

## Antibodies

Antibodies used

All antibodies have been described as required in Methods.
TβRII (sc-400, Santa Cruz Biotechnology, 1:1000 for IB, 1:50 for IP, 1:100 for IHC, 1:50 for immunogold label, 1:100 for IF), human TβRII (ab184948, Abcam, 1:1000 for IB), CD63 (ab216130, Abcam, 1:2000 for IB), TSG101 (sc-7964, Santa Cruz Biotechnology, 1:1000 for IB), Alix (sc-53540, Santa Cruz Biotechnology, 1:1000 for IB, 1:100 for IF), CD9 (A19027, ABclonal, 1:2000 for IB), CD81(sc-166029, Santa Cruz Biotechnology, 1:1000 for IB), Calnexin (A4846, ABclonal, 1:2000 for IB), Hrs (A1790, ABclonal, 1:2000 for IB), Rab27a (sc-74586, Santa Cruz Biotechnology, 1:1000 for IB), N-cadherin (610920, BD Bioscience, 1:50000 for IB), E-cadherin (610181, BD Bioscience, 1:10000 for IB, 1:100 for IF), SMAD4 (sc-7966, Santa Cruz Biotechnology, 1:1000 for IB), SMAD2-3 (610842, BD Bioscience, 1:2500 for IB, 1:500 for IP, 1:100 for IF), phospho-SMAD2 (#3101, Cell Signaling, 1:5000 for IB, 1:50 for IHC), SMAD3 (A19115, ABclonal, 1:100 for IP, 1:2000 for IB), phosphor-SMAD3 (AP0727, ABclonal, 1:2000 for IB), SMAD4 (A19116, ABclonal, 1:2000 for IB), Ub (sc-8017, Santa Cruz Biotechnology, 1:1000 for IB), fibronectin (SAB4500974, Sigma, 1:1000 for IB), SMA (#SAB5500002, Sigma, 1:1000 for IB), vimentin (#5741, Cell Signaling, 1:1000 for IB), TCF1/7 (#2203, Cell Signaling, 1:1000 for IB), EOMES (#4540, Cell Signaling, 1:1000 for IB), GZMB (sc-8022, Santa Cruz Biotechnology, 1:1000 for IB), CD8a (ab22378, Abcam, 1:400 for IHC), K48-linkage specific polyubiquitin (#8081S, Cell Signaling, 1:1000 for IB), K63-linkage specific polyubiquitin (#A18164, ABclonal, 1:1000 for IB), β-actin (#A5441, Sigma, 1:10000 for IB), Phalloidin (#93042, Sigma, 1:1000 for IF), polyclonal HA(Y-11) (sc-805, Santa Cruz Biotechnology, 1:1000 for IB), monoclonal HA(12CAS5, home-made, 1:5000 for WB), polyclonal Myc (A-14) (sc-789, Santa Cruz Biotechnology, 1:1000 for IB), monoclonal Myc (9E10) (sc-40, Santa Cruz Biotechnology, 1:1000 for IB), Flag (M2, Sigma, 1:10000 for IB, 1:200 for IF), Protein A–HRP (Sigma-Aldrich GENA9120, 1:10000 for IB), HRP-conjugated secondary antibodies to mouse (NA931) or rabbit (NA934) (both from Amersham Biosciences, 1:10000 for IB), AlexaFluor488-labeled secondary antibody to rabbit (Molecular Probes R37116, 1:300 for IF) or AlexaFluor593-labeled antibody to mouse (Molecular Probes R3712, 1:300 for IF). TCF1 (Cell Signaling, #2203S, 1:50), SMAD3 (Cell Signaling, #9523S, 1:50) were used for ChIP-qPCR. Anti-CD3e PE (#553063, clone 145-2C11, 1:100, BD), anti-CD4 FITC (#11-0042-82, clone RM4-5, 1:100, eBioscience), anti-CD8 APC (#100712, clone 53-6.7, 1:100, Biolgend), anti-Ly-6G FITC (#11-5931-82, clone RB6-8C5, 1:100, eBioscience), anti-CD11b PE (#12-0112-82, clone M1/70, 1:100, eBioscience), anti-F4/80 FITC (#11-4801-82, clone BM8, 1:100, eBioscience), anti-PD1 FITC (#11-9981-82, clone RMP1-30, 1:100, eBioscience), anti-LAG-3 FITC (#11-2231-82, clone C9B7W, 1:50, eBioscience), anti-TIM3 FITC (#11-5870-82, clone RMT3-23, 1:100, eBioscience), anti-IFN-γ PE (#12-7311-81, clone XMG1.2, 1:100, eBioscience), anti-TNFα FITC (#11-7321-82, clone MP6-XT22, 1:100, eBioscience), anti-FOXP3 PE (#12-5773-82, clone FJK-16s, 1:50, eBioscience), anti-GZMB FITC (#372206, clone QA16A02, 1:50, Biolgend) were used for FACS. Primary antibodies to p-SMAD2 (1:50; Cell Signaling, #3108) was used for immunohistochemical stainings. Anti-CD8α (clone 53-6.7, #BE0004-1, Bio X Cell) or anti-PD-1 antibody (clone J43, #BP0033-2, Bio X Cell) was used for mice assay. Anti-human PD1 antibody (clone J116, #BE0188, BioXcell) or anti-human PD-L1 antibody (clone 29E.2A3, #BE0285, BioXcell) was used for T Cell-Mediated Tumor Cell Killing Assay.

Validation

All antibodies used in our study have been validated and detailed information could be found on the website from manufactures as listed below. Additional information on validation can be found on the manufacturers' websites.
Anti-Flag, anti-Myc and anti-HA antibodies were validated using cell lysates containing overexpressed Flag-, Myc or HA-tagged proteins.

TβRII (sc-400, Santa Cruz Biotechnology), WB, IP, IF, IHC(P), ELISA/ Mouse, Rat, Human/ Rabbit polyclonal, https://www.scbt.com/p/tgfbeta-rii-antibody-l-21?requestFrom=search

TβRII (ab184948, Abcam), WB/Human/ Rabbit monoclonal, https://www.abcam.cn/tgf-beta-receptor-ii-antibody-epr14673-ab184948.html

CD63 (ab216130, Abcam), IHC-P, WB/ Mouse, Human/ Rabbit polyclonal, https://www.abcam.cn/cd63-antibody-late-endosome-marker-ab216130.html

TSG101 (sc-7964, Santa Cruz Biotechnology), WB, IP, IF, IHC(P)/ mouse, rat, human/ Mouse monoclonal, https://www.scbt.com/p/tsg-101-antibody-c-2?requestFrom=search

Alix (sc-53540, Santa Cruz Biotechnology), WB, IP, IF, IHC(P), ELISA/ mouse, rat, human/ Mouse monoclonal, https://www.scbt.com/p/alix-antibody-1a12?requestFrom=search

CD9 (A19027, ABclonal), WB, IP, IHC / mouse, human/ Rabbit monoclonal, https://abclonal.com.cn/catalog/A19027

CD81(sc-166029, Santa Cruz Biotechnology), WB, IP, IF, IHC(P), ELISA/ mouse, rat, human/ Mouse monoclonal, https://www.scbt.com/p/cd81-antibody-b-11?requestFrom=search

Calnexin (A4846, ABclonal), WB/ human/ Rabbit monoclonal, https://abclonal.com.cn/catalog/A4846

Hrs (A1790, ABclonal), WB, IF/ mouse, rat, human/ Rabbit polyclonal, https://abclonal.com.cn/catalog/A1790

Rab27a (sc-74586, Santa Cruz Biotechnology), WB, IP, IF, IHC(P), ELISA/ mouse, rat, human/ Mouse monoclonal, https://www.scbt.com/p/rab-27a-antibody-e-8?requestFrom=search

N-cadherin (610920, BD Bioscience), WB, IP, IF/ mouse, rat, human, chicken/ Mouse monoclonal, https://www.bdbiosciences.com/zh-cn/products/reagents/microscopy-imaging-reagents/immunofluorescence-reagents/purified-mouse-anti-n-cadherin.610920

E-cadherin (610181, BD Bioscience), WB, IP, IF, IHC/ mouse, rat, human, dog/ Mouse monoclonal, https://www.bdbiosciences.com/zh-cn/products/reagents/microscopy-imaging-reagents/immunofluorescence-reagents/purified-mouse-anti-e-cadherin.610181

SMAD4 (sc-7966, Santa Cruz Biotechnology), WB, IP, IF, IHC(P), FCM, ELISA/ mouse, rat, human/ Mouse monoclonal, https://www.scbt.com/p/smad4-antibody-b-8?requestFrom=search

SMAD2-3 (610842, BD Bioscience), WB, IP, IF / mouse, rat, human, dog/ Mouse monoclonal, https://www.bdbiosciences.com/zh-cn/products/reagents/microscopy-imaging-reagents/immunofluorescence-reagents/purified-mouse-anti-smad2-3.610842
phospho-SMAD2 (#3101, Cell Signaling), WB/ mouse, rat, human/ Rabbit polyclonal, https://www.cellsignal.cn/products/primary-antibodies/phospho-smad2-ser465-467-antibody/3101?site-search-type=Products&N=4294956287&Ntt=3101&fromPage=plp&_requestid=3780032

SMAD3 (A19115, ABclonal), WB, IHC, IF, IP/ Mouse, Rat, Human/ Rabbit monoclonal, https://abclonal.com.cn/catalog/A19115

phosphor-SMAD3 (AP0727, ABclonal), WB, IHC/ Mouse, Rat, Human/ Rabbit monoclonal, https://abclonal.com.cn/catalog/AP0727

SMAD4 (A19116, ABclonal), WB, IHC, IP/ Mouse, Rat, Human/ Rabbit monoclonal, https://abclonal.com.cn/catalog/A19116

Ub (sc-8017, Santa Cruz Biotechnology), WB, IHC(P), IP, IF, FCM, ELISA/ Mouse, Rat, Human, Drosophila/ Mouse monoclonal, https://www.scbt.com/p/ubiquitin-antibody-p4d1?requestFrom=search

fibronectin (SAB4500974, Sigma), WB, IHC, ELISA/ Mouse, Rat, Human/ Rabbit polyclonal, https://www.sigmaaldrich.cn/CN/zh/product/sigma/sab4500974

SMA (#SAB5500002, Sigma), WB, IHC/ Human/ Rabbit monoclonal, https://www.sigmaaldrich.cn/CN/zh/product/sigma/sab5500002

vimentin (#5741, Cell Signaling), WB, IHC, IF, FCM/ Mouse, Rat, Human, Monkey/ Rabbit monoclonal, https://www.cellsignal.cn/products/primary-antibodies/vimentin-d21h3-xp-rabbit-mab/5741?site-search-type=Products&N=4294956287&Ntt=5741&fromPage=plp&_requestid=3785802

TCF1/7 (#2203, Cell Signaling), WB, IP, IHC, IF, FCM, ChIP/ Mouse, Human/ Rabbit monoclonal, https://www.cellsignal.cn/products/primary-antibodies/tcf1-tcf7-c63d9-rabbit-mab/2203?site-search-type=Products&N=4294956287&Ntt=2203&fromPage=plp&_requestid=3787125

EOMES (#4540, Cell Signaling), WB/ Mouse/ Rabbit polyclonal, https://www.cellsignal.cn/products/primary-antibodies/eomes-antibody/4540?site-search-type=Products&N=4294956287&Ntt=4540&fromPage=plp&_requestid=3788096

GZMB (sc-8022, Santa Cruz Biotechnology), WB, IP, IHC(P), IF, ELISA/ Mouse, Human, Rat/ Mouse monoclonal, https://www.scbt.com/p/granzyme-b-antibody-2c5?requestFrom=search

CD8a (ab22378, Abcam), Flow Cyt/ Mouse/ Rat monoclonal, https://www.abcam.cn/cd8-alpha-antibody-yts1694-ab22378.html

K48-linkage specific polyubiquitin (#8081S, Cell Signaling), WB/ All/ Rabbit monoclonal, https://www.cellsignal.cn/products/primary-antibodies/k48-linkage-specific-polyubiquitin-d9d5-rabbit-mab/8081?site-search-type=Products&N=4294956287&Ntt=8081s&fromPage=plp&_requestid=3790533

K63-linkage specific polyubiquitin (#A18164, ABclonal), WB/ Mouse, Human, Rat/ Rabbit polyclonal, https://abclonal.com.cn/catalog/A18164

β-actin (#A5441, Sigma), WB, IHC, ELISA, IF/ pig, Hirudo medicinalis, bovine, rat, canine, feline, human, rabbit, carp, mouse, guinea pig, chicken, sheep/ Mouse monoclonal, https://www.sigmaaldrich.cn/CN/zh/product/sigma/a5441

polyclonal HA(Y-11) (sc-805, Santa Cruz Biotechnology), WB, ELISA, IP, IF, FCM/ Mouse monoclonal, https://www.scbt.com/p/ha-probe-antibody-y-11?requestFrom=search

TCF1 (Cell Signaling, #2203S), WB, IHC, IP, IF, FCM,ChIP/ Mouse, Human/ Rabbit monoclonal, https://www.cellsignal.cn/products/primary-antibodies/tcf1-tcf7-c63d9-rabbit-mab/2203?site-search-type=Products&N=4294956287&Ntt=2203s&fromPage=plp&_requestid=3795662

SMAD3 (Cell Signaling, #9523S), WB, IP, IF, FCM,ChIP/ Mouse, Human, Rat, Monkey/ Rabbit monoclonal, https://www.cellsignal.cn/products/primary-antibodies/smad3-c67h9-rabbit-mab/9523?site-search-type=Products&N=4294956287&Ntt=9523s&fromPage=plp&_requestid=3796303

Anti-CD3e PE (#553063, clone 145-2C11, BD), Flow Cyt/ Mouse/ Hamster monoclonal, https://www.bdbiosciences.com/zh-cn/products/reagents/flow-cytometry-reagents/research-reagents/single-color-antibodies-ruo/pe-hamster-anti-mouse-cd3e.553063

anti-CD4 FITC (#11-0042-82, clone RM4-5, eBioscience), Flow Cyt/ Mouse/ Rat monoclonal, https://www.fishersci.com/shop/products/cd4-rat-anti-mouse-fitc-clone-rm4-5-ebioscience-3/501129681?searchHijack=true&searchTerm=11-0042-82&searchType=RAPID&matchedCatNo=11-0042-82

anti-CD8 APC (#100712, clone 53-6.7, Biolgend), Flow Cyt/ Mouse/ Rat monoclonal, https://www.biolegend.com/en-us/products/apc-anti-mouse-cd8a-antibody-150

anti-Ly-6G FITC (#11-5931-82, clone RB6-8C5, eBioscience), Flow Cyt/ Mouse/ Rat monoclonal, https://www.fishersci.com/shop/products/ly-6g-gr-1-rat-anti-mouse-fitc-clone-rb6-8c5-ebioscience-2/501129689?searchHijack=true&searchTerm=11-5931-82&searchType=RAPID&matchedCatNo=11-5931-82

anti-CD11b PE (#12-0112-82, clone M1/70, eBioscience), Flow Cyt/ Mouse/ Rat monoclonal, https://www.fishersci.com/shop/products/cd11b-rat-anti-mouse-pe-clone-m1-70-ebioscience-2/501129669?searchHijack=true&searchTerm=12-0112-82&searchType=RAPID&matchedCatNo=12-0112-82

anti-F4/80 FITC (#11-4801-82, clone BM8, eBioscience), Flow Cyt/ Mouse/ Rat monoclonal, https://www.fishersci.com/shop/

products/f4-80-antigen-rat-anti-mouse-fitc-clone-bm8-ebioscience-2/501129010?searchHijack=true&searchTerm=11-4801-82&searchType=RAPID&matchedCatNo=11-4801-82

anti-PD1 FITC (#11-9981-82, clone RMP1-30, eBioscience), Flow Cyt/ Mouse/ Rat monoclonal, https://www.fishersci.com/shop/products/cd279-pd-1-rat-anti-mouse-fitc-clone-rmp1-30-ebioscience-2/5010106?searchHijack=true&searchTerm=11-9981-82&searchType=RAPID&matchedCatNo=11-9981-82

anti-LAG-3 FITC (#11-2231-82, clone C9B7W, eBioscience), Flow Cyt/ Mouse/ Rat monoclonal, https://www.fishersci.com/shop/products/cd223-lag-3-rat-anti-mouse-fitc-clone-ebioc9b7w-c9b7w-ebioscience-1/501129766?searchHijack=true&searchTerm=11-2231-82&searchType=RAPID&matchedCatNo=11-2231-82

anti-TIM3 FITC (#11-5870-82, clone RMT3-23, eBioscience), Flow Cyt/ Mouse/ Rat monoclonal, https://www.fishersci.com/shop/products/cd366-tim3-fitc-clone-rmt3-23-ebioscience-2/11587082?searchHijack=true&searchTerm=11-5870-82&searchType=RAPID&matchedCatNo=11-5870-82

anti-IFN-γ PE (#12-7311-81, clone XMG1.2, eBioscience), Flow Cyt/ Mouse/ Rat monoclonal, https://www.fishersci.com/shop/products/ifn-gamma-rat-anti-mouse-pe-clone-xmg1-2-ebioscience-3/5011184?searchHijack=true&searchTerm=12-7311-81&searchType=RAPID&matchedCatNo=12-7311-81

anti-TNFα FITC (#11-7321-82, clone MP6-XT22, eBioscience), Flow Cyt/ Mouse/ Rat monoclonal, https://www.fishersci.com/shop/products/tnf-alpha-rat-anti-mouse-fitc-clone-mp6-xt22-ebioscience-3/5010033?searchHijack=true&searchTerm=11-7321-82&searchType=RAPID&matchedCatNo=11-7321-82

anti-FOXP3 PE (#12-5773-82, clone FJK-16s, eBioscience), Flow Cyt/ Mouse, Bovine, Canine, Feline, Porcine, Rat/ Rat monoclonal, https://www.fishersci.com/shop/products/foxp3-rat-anti-mouse-rat-pe-clone-fjk-16s-ebioscience-2/501129663?searchHijack=true&searchTerm=12-5773-82&searchType=RAPID&matchedCatNo=12-5773-82

anti-GZMB FITC (#372206, clone QA16A02, Biolgend), ICFC/ Human, Mouse/ Mouse Recombinant, https://www.biolegend.com/en-us/products/fitc-anti-human-mouse-granzyme-b-recombinant-antibody-14430

p-SMAD2 (#3108, Cell Signaling), WB/ Human, Mouse, Rat, Mink/ Rabbit monoclonal, https://www.cellsignal.cn/products/primary-antibodies/phospho-smad2-ser465-467-138d4-rabbit-mab/3108?site-search-type=Products&N=4294956287&Ntt=3108&fromPage=plp&_requestid=3829713

Anti-CD8α (clone 53-6.7, #BE0004-1, BioXCell), WB, IF, Flow Cyt, in vivo CD8+ T cell depletion/ Mouse/ Rat monoclonal, https://bxcell.com/product/m-cd8a/

anti-PD-1 antibody (clone J43, #BP0033-2, BioXCell), WB, in vivo blocking of PD-1/PD-L signaling, in vitro PD-1 neutralization/ Mouse/ Hamster polyclonal, https://bxcell.com/product/invivoplus-anti-m-pd-1-cd279/

Anti-human PD1 antibody (clone J116, #BE0188, BioXcell), in vivo PD-1 blockade in humanized mice, in vitro PD-1 neutralization/ Human/ Mouse monoclonal, https://bxcell.com/product/h-cd279-pd-1/

anti-human PD-L1 antibody (clone 29E.2A3, #BE0285, BioXcell), in vitro PD-1 blockade, IHC, Flow Cyt/ Human/ Mouse monoclonal, https://bxcell.com/product/invivomab-anti-human-pd-l1-b7-h1/

## Eukaryotic cell lines

Policy information about cell lines

| | |
|---|---|
| Cell line source(s) | HEK293T, HeLa, DR26, HaCaT, MCF7, MDA-MB-231, 4T07 and 4T1 cells were originally from ATCC; MCF10A (MI); MCF10A-RAS (MII) cell lines were obtained from Dr. Fred Miller (Barbara Ann Karmanos Cancer Institute, Detroit, USA) |
| Authentication | Cell lines were not authenticated. |
| Mycoplasma contamination | We confirm that all cell lines were negative for mycoplasma contamination. |
| Commonly misidentified lines (See ICLAC register) | No commonly misidentified cell lines were used in this study. |

## Animals and other organisms

Policy information about studies involving animals; ARRIVE guidelines recommended for reporting animal research

| | |
|---|---|
| Laboratory animals | Five-weeks-old nude mice or female syngeneic BALB/c mice were purchased from the animal husbandry center of the Shanghai Institute of Cell Biology, Academia Sinica, Shanghai, China. Details of animals and animal-derived materials were described in Methods. Mice were maintained under specific-pathogen-free conditions in the animal facility of Soochow University. The animal room has a controlled temperature (18-23°C), humidity (40-60%), and a 12 light/12 dark cycle. |
| Wild animals | No wild animals were used in this study. |
| Field-collected samples | No field-collected samples were used in this study. |

Ethics oversight

All procedures in this study involving animals were reviewed and approved by the Committee for Animal Welfare in Soochow University.

Note that full information on the approval of the study protocol must also be provided in the manuscript.

## Human research participants

Policy information about studies involving human research participants

Population characteristics

Blood samples from healthy donors and patients with breast cancer were obtained from the First Affiliated Hospital of Zhejiang University and Zhejiang Provincial People's Hospital after providing informed consent. 46 breast cancer patients were diagnosed as Luminal A (n=6), Luminal B (n=7), HER2+ (n=13), TNBC (n=20).The clinical characteristics of each patient are provided in Supplementary Table 1.

Recruitment

Samples for banking and for the in vitro colony studies were obtained from breast cancer patients seen for evaluation, follow-up, and/or treatment in the clinics of the respective institutions, after informed consent was obtained. We then received de-identified samples, selected randomly and unbiased, from the tumor banks. Participants did not receive any compensation.

Ethics oversight

Samples were collected under the approved sample collection protocol by The First Affiliated Hospital of Zhejiang University and Zhejiang Provincial People's Hospital Research Ethics Committee.

Note that full information on the approval of the study protocol must also be provided in the manuscript.

## Flow Cytometry

### Plots

Confirm that:

☒ The axis labels state the marker and fluorochrome used (e.g. CD4-FITC).

☒ The axis scales are clearly visible. Include numbers along axes only for bottom left plot of group (a 'group' is an analysis of identical markers).

☒ All plots are contour plots with outliers or pseudocolor plots.

☒ A numerical value for number of cells or percentage (with statistics) is provided.

### Methodology

Sample preparation

Lymphocytes from the spleen or lymph nodes were depleted of erythrocytes by hypotonic lysis. To obtain tumor-infiltrating lymphocytes (TIL), excised tumors were harvested, cut into 1mm3 with scissors and digested with 5 ml tumour digestion buffer (5% FBS, 1 mg/ml collagenase IV and 100 µg/mL DNase I). Tissues were digested after rotation for 1 h at 37°C using the gentle MACS Dissociator (Miltenui Biotec Inc., San Diego, CA, USA). The cell suspension was filtered using a 70-µm filter to obtain a single-cell suspension. TILs were enriched on a Ficoll gradient (Sigma) and washed with DMEM supplemented with 10% FBS.
For the intracellular cytokine staining, lymphocytes were first stained with anti-CD3e PE (553063, BD), anti-CD4 FITC (eBioscience, 11-0042-82), anti-CD8 APC (100712, Biolgend), anti-Ly-6G FITC (11-5931-82, eBioscience), anti-CD11b PE (12-0112-82, eBioscience), anti-F4/80 FITC (11-4801-82, eBioscience) antibodies, anti-PD1 FITC (11-9981-82, RMP1-30, eBioscience), anti-LAG-3 FITC (11-2231-82, eBioscience), anti-TIM3 FITC (11-5870-82, RMT3-23, eBioscience) fixed and permeabilized with a Cytofix/Cytoperm kit (554722, BD Biosciences PharMingen) or Foxp3/Transcription Factor Staining Buffer Set (00-5523-00, eBioscience), and finally stained with anti-IFN-γ PE (12-7311-81), anti-TNFα FITC (11-7321-82, MP6-XT22, eBioscience), anti-FOXP3 PE (12-5773-82), anti-GZMB FITC (372206, Biolgend) antibodies in accordance with the manufacturer's instructions.
For the analysis of EVs, 20 µg EVs were mixed with 5 µl 4 µm aldehyde/sulphate latex beads (Invitrogen, no.1743119) in 500 µl 1 x PBS for 30 min at room temperature with continuous rotation. EVs-bound beads were incubated with 1 µg anti-TβRII APC-conjugated antibody (no.FAB2411A, R&D) for 30 min.

Instrument

All data were collected with Beckman CytoFlex (Beckman).

Software

CytExpert Software and FlowJo (TreeStar) were used for data collection and analyzing.

Cell population abundance

For flow sorting, post-sort cells were analyzed by flow cytometry and the purity was at least 95%. For MACS sorting, post-sort cells were at least 95% pure for CD8 T cells.

Gating strategy

FSC SSC to remove debris; SSC-H by SSC-A to define single cells. Positive populations
were defined using not stained cells as reference. Isotype controls were used to confirm the specificity of the staining. All the gating strategy used for all relevant experiments were shown in Supplementary Figure 5a.

☒ Tick this box to confirm that a figure exemplifying the gating strategy is provided in the Supplementary Information.
